# Histamine therapeutic efficacy in metastatic melanoma: Role of histamine H_4_ receptor agonists and opportunity for combination with radiation

**DOI:** 10.18632/oncotarget.15594

**Published:** 2017-02-21

**Authors:** Noelia A. Massari, Melisa B. Nicoud, Lorena Sambuco, Graciela P. Cricco, Diego J. Martinel Lamas, María V. Herrero Ducloux, Horacio Blanco, Elena S. Rivera, Vanina A. Medina

**Affiliations:** ^1^ Laboratory of Radioisotopes, School of Pharmacy and Biochemistry, University of Buenos Aires, Buenos Aires, Argentina; ^2^ Immunology Department, School of Natural Sciences, National University of Patagonia San Juan Bosco, Chubut, Argentina; ^3^ Laboratory of Tumor Biology and Inflammation, Institute for Biomedical Research (BIOMED), School of Medical Sciences, Pontifical Catholic University of Argentina (UCA), and the National Scientific and Technical Research Council (CONICET), Buenos Aires, Argentina; ^4^ Institute of Immunooncology, Buenos Aires, Argentina; ^5^ Pathology Department, School of Natural Sciences, National University of Patagonia San Juan Bosco, Chubut, Argentina; ^6^ Hospital Municipal de Oncología “Marie Curie”, Buenos Aires, Argentina

**Keywords:** H_4_R, JNJ28610244, experimental melanoma, tumor growth, ionizing radiation

## Abstract

The aims of the work were to improve our knowledge of the role of H_4_R in melanoma proliferation and assess *in vivo* the therapeutic efficacy of histamine, clozapine and JNJ28610244, an H_4_R agonist, in a preclinical metastatic model of melanoma. Additionally, we aimed to investigate the combinatorial effect of histamine and gamma radiation on the radiobiological response of melanoma cells.

Results indicate that 1205Lu metastatic melanoma cells express H_4_R and that histamine inhibits proliferation, in part through the stimulation of the H_4_R, and induces cell senescence and melanogenesis. Daily treatment with H_4_R agonists (1 mg/kg, *sc*) exhibited a significant *in vivo* antitumor effect and importantly, compounds reduced metastatic potential, particularly in the group treated with JNJ28610244, the H_4_R agonist with higher specificity. H_4_R is expressed in benign and malignant lesions of melanocytic lineage, highlighting the potential clinical use of histamine and H_4_R agonists. In addition, histamine increased radiosensitivity of melanoma cells *in vitro* and *in vivo*. We conclude that stimulation of H_4_R by specific ligands may represent a novel therapeutic strategy in those tumors that express this receptor. Furthermore, through increasing radiation-induced response, histamine could improve cancer radiotherapy for the treatment of melanoma.

## INTRODUCTION

The incidence and mortality of human melanoma continues increasing progressively, particularly in patients who have developed the most severe forms (e.g. stage IV) or metastatic melanoma. These facts make this disease a priority health issue, given the high resistance to existing treatments such as chemotherapy, immunotherapy, or even targeted therapies with monoclonal antibodies. Oncogene-targeted therapy and immune checkpoint blockade have shown notable efficacy in a subset of melanoma patients with some clinical impact. However, the study of new approaches and therapeutic targets is urgently needed considering the problems associated with highly toxic therapies, elevated costs and the fact that most patients show a disease-free interval of only a few months [1, 2, 3, 4].

Histamine is involved in numerous physiological and pathological conditions of skin [5, 6, 7]. Although there are several studies describing the action of histamine as a modulator of proliferation of certain tumors, its role in malignant melanoma is still inconclusive [5, 8, 9, 10]. It seems that histamine effect on tumor proliferation depends on the cell type, the balance between different receptor subtypes expressed, the concentration of histamine reached in the tumoral environment and the effectors activated, among others.

On one side, the use of specific anti-sense oligonucleotides against histidine decarboxylase (HDC) decreased the proliferation rate of human melanoma cells [11], while over-expression of HDC markedly accelerates tumor growth and increases metastatic colony-forming potential in mouse melanoma [12, 13]. On the other side, it was demonstrated by using histamine agonists, antagonists and genetic tools, that histamine treatment produced an inhibitory effect on proliferation mediated in part through the stimulation of the H_4_R in WM35 and M1/15 human melanoma cells. *In vivo* experiments on M1/15 human primary melanoma experimental model demonstrated that mice receiving histamine or clozapine (H_4_R agonist) showed an increased median survival associated to a decrease in tumor growth and intratumoral neovascularization [8, 9]. In line with these results, numerous phase II and III clinical trials in metastatic melanoma demonstrated clinical benefits of histamine (Ceplene, a synthetic derivative of histamine) as an adjuvant to immunotherapy with IL-2, especially in melanoma patients with liver metastases [14]. Histamine dihydrochloride inhibits the formation of reactive oxygen species from monocytes/macrophages by suppressing the activity of NADPH oxidase, and thus preventing the inactivation of T cells and NK cells [15]. In addition, it is not possible to discard a direct action of histamine on melanoma cells, considering that the expression of H_1_R, H_2_R, H_3_R and H_4_R in human melanoma cell lines was shown [8, 12, 16]. In addition, literature suggests that those with allergy have a reduced risk of developing cancer versus the general population [17] and that a history of asthma may be a protective factor in cutaneous melanoma [18].

Based on the presented evidence, the aim of this work was to improve our knowledge of the role of H_4_R in melanoma proliferation and assess *in vivo* the therapeutic efficacy of histamine, clozapine and JNJ28610244, a new compound with excellent selectivity and high affinity for human H_4_R, in a preclinical metastatic model of melanoma. In addition, we aimed to investigate the combinatorial effect of histamine and gamma radiation *in vitro* and *in vivo* on the radiobiological response of melanoma cells. The tumorigenic and highly invasive malignant 1205Lu human melanoma cell line was used for these purposes [19, 20].

## RESULTS

### Role of H_4_R in human 1205Lu melanoma cell proliferation, differentiation and senescence

**Figure 1:**
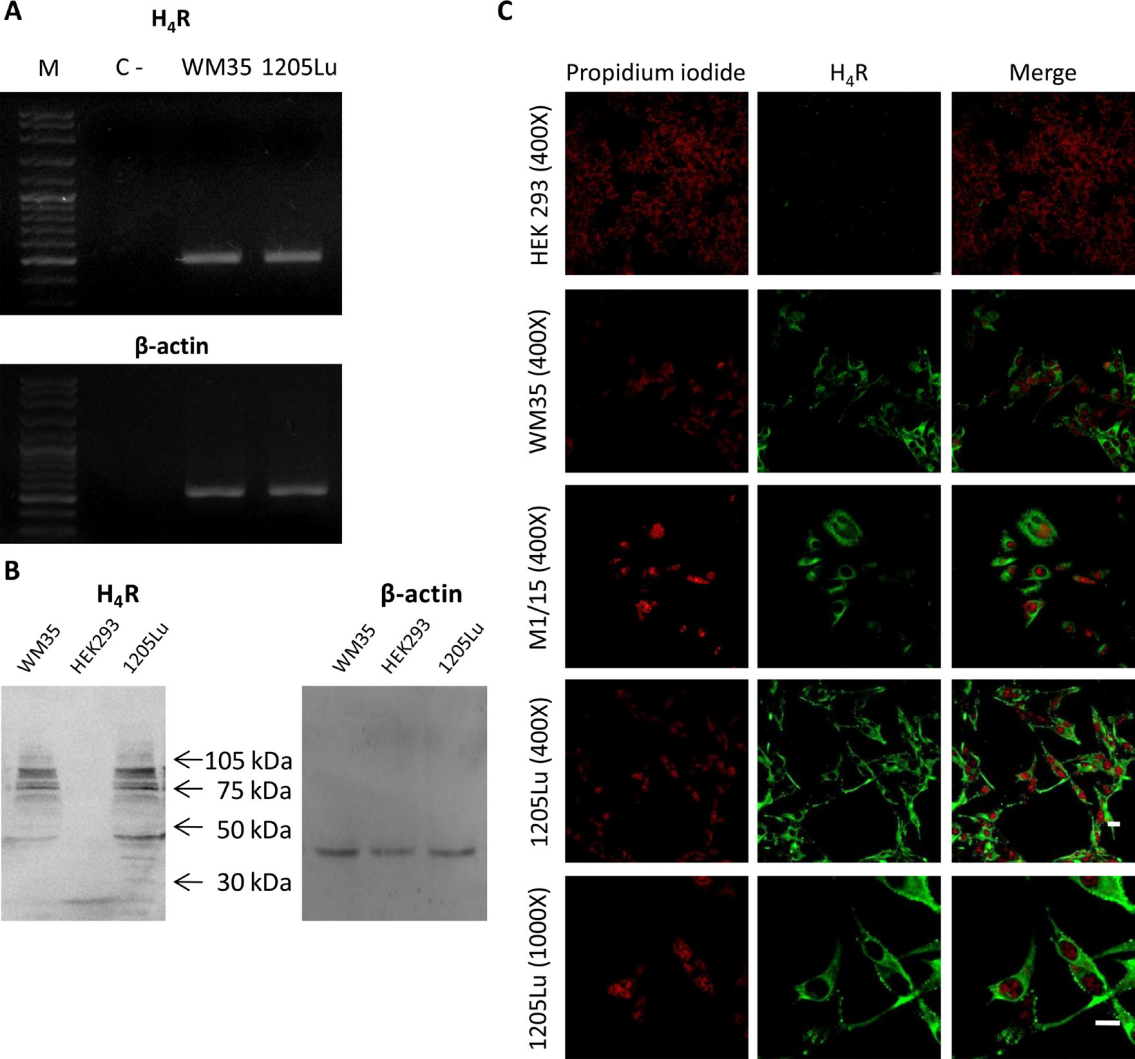
H_4_R expression in 1205Lu cells H_4_R receptor expression was determined by RT-PCR, Western blot and Immunofluorescence. (**A**) RT-PCR of H_4_R. Lanes: M, DNA ladder molecular size marker; WM35, human primary melanoma cells were used as positive control; 1205Lu, human metastatic melanoma cells. β-actin (521 bp) was used as load control. (**B**) Western blot of H_4_R. WM35 cells were used as positive control. HEK293 cells were used as negative control. β-actin (42 kDa) was used as load control. (**C**) Immunofluorescence (green) of H_4_R in 1205Lu cells evaluated by confocal microscopy. Nuclei were counterstained with ethidium bromide (red). Pictures were taken at 400X-fold and 1000X-fold magnification. Scale bar = 20 μm. Representative results of three independent experiments. WM35 and M1/15 cells were used as positive control. HEK293 cells were used as negative control.

We first evaluated the expression of H_4_R in 1205Lu malignant melanoma cells. Figure 1A shows that 1205Lu cell line expressed the H_4_R at the mRNA level. The identity of H_4_R was confirmed by sequencing and protein expression of H_4_R was further demonstrated by Western blot (Figure 1B).

Western blot analysis demonstrated the presence of a diverse range of molecular weight species of the H_4_R, which are in agreement with previous reports in several cell lines, including melanoma cells [8, 21, 22, 23].

The presence of H_4_R in 1205Lu cells was verified by immunostaining and confocal microscopy (Figure 1C). The specificity of H_4_R antibody was evaluated by immunofluorescence and Western blot analysis, using WM35 and M1/15 melanoma cell lines as positive controls [8] and HEK293 cell line as a negative control of H_4_R expression [24], (Figure 1B, 1C). Furthermore, siRNA specific for H_4_R mRNA was used to knock down its expression in melanoma cells, which was ascertained by immunocytochemistry (Supplementary Figure 1 of Supplementary Data).

**Figure 2:**
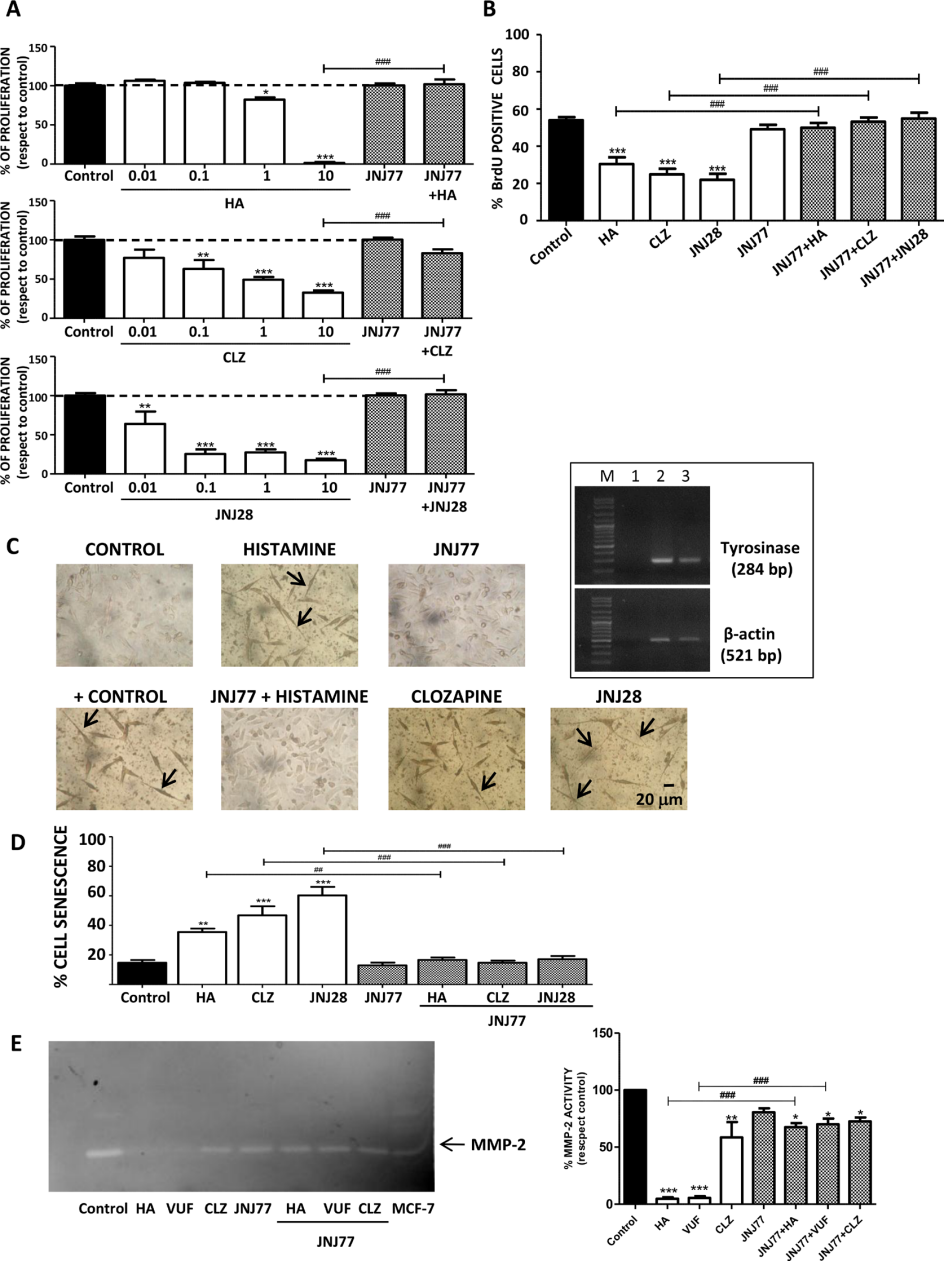
H_4_R-induced biological responses in 1205Lu cells Cells were left untreated (control) or treated with histamine (HA), clozapine (CLZ), JNJ28610244 (JNJ28) or VUF8430 (VUF) and/or 10 μM JNJ7777120 (JNJ77) (**A**) Clonogenic assay. (**B**) Incorporation of BrdU-positive cells assessed by immunocytochemistry 48 h after treatment. (**C**) L-dopa staining with melanin precursor (10 mM L-dopa). evaluated 10 days after treatment. Localization of dopa-oxidase was indicated by the presence of an insoluble brown/black precipitate. Pictures were taken at 200X-fold magnification. Scale bar = 20 μm. Arrows indicate cell prolongations. Positive control: 16 nM TPA + L-dopa. Inset: Tyrosinase expression in 1205Lu was evaluated by RT-PCR (284 bp). Lanes: M, DNA ladder molecular size marker; 1, Negative control (without cDNA); 2: Positive control (M1/15 human melanoma cells), 3: 1205Lu cells. β-actin (521 bp) was used as load control. (**D**) Senescence-associated to β-galactosidase staining evaluated 48 h after treatment. (**E**) MMP-2 gelatinololytic activity determined 24 h after treatment, MCF-7 cells were used as positive control. Error bars represent the means ± SEM of three independent experiments (ANOVA and Dunnett's Multiple Comparison Test, *P < 0.05, **P < 0.01, ***P < 0.001 vs. control; ANOVA and Newman–Keuls Multiple Comparison Test, ^##^*P* < 0.01, ^###^*P* < 0.001 vs. JNJ77+HA or H_4_R agonist). Results are representative of three independent experiments.

Results demonstrate that histamine and H_4_R agonists significantly decreased clonogenic proliferation of human melanoma cells (IC_50_= 1.6 μM; 0.7 μM; 1 μM for histamine, clozapine and JNJ28610244, respectively), effect that was blocked with the combined treatment with the H_4_R antagonist JNJ7777120 (Figure 2A). The inhibitory effect of H_4_R on proliferation was confirmed by assessing the incorporation of BrdU, a thymidine analog. Histamine and both H_4_R agonists significantly reduced the incorporation of BrdU in 1205Lu cells. Treatment with JNJ7777120, added 30 minutes before any other treatment, completely reversed the effect of the H_4_R ligands on melanoma cells (Figure 2B).

The induction of cell differentiation and/or senescence was then investigated. 1205Lu cell line expressed tyrosinase mRNA, the rate-limiting enzyme in melanin production (Figure 2C, inset). The oxidation of L-dopa test revealed that treatment with H_4_R agonists stimulated melanogenesis *in vitro*. Synthesis of melanin was detected as a brown-black pigmentation in cells under light microscopy (Figure 2C). In addition, changes in cell morphology, as extensions or cell prolongations, were also observed and these characteristics are associated with terminal cell differentiation process [25, 26] (Figure 2C).

On the other hand, results demonstrated that treatment with histamine and H_4_R agonists significantly increased the percentage of senescent cells evidenced by an enhanced activity of senescence associated β-galactosidase (histamine: 35.5 % ± 2.3 %; clozapine: 46.8 % ± 6.2 %; JNJ28610244: 60.3 % ± 5.7 % vs. control: 14.7 % ± 1.9 %) (Figure 2D). The combined treatment with the H_4_R antagonist reversed the increase in cell senescence and differentiation, reinforcing the important role of H_4_R in these cellular events (Figure 2C, 2D).

### Effect of histamine, clozapine and JNJ28610244 on human melanoma tumors developed in nude mice

**Figure 3:**
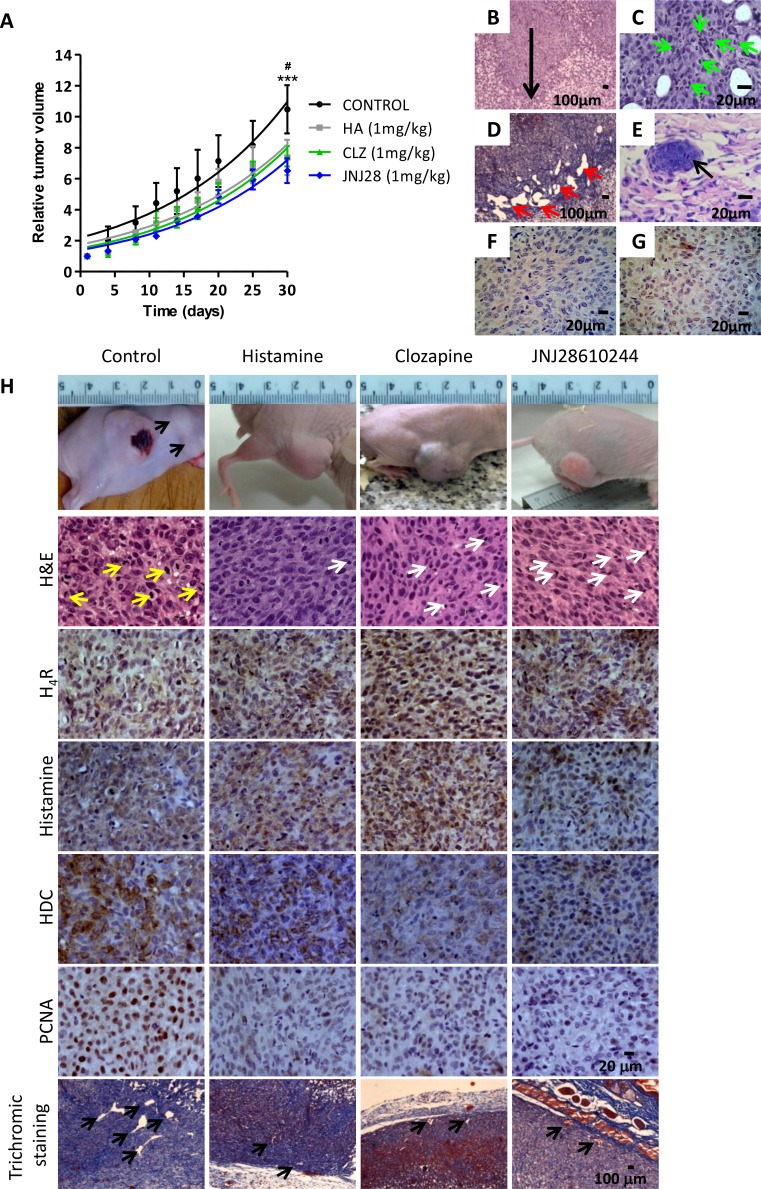
Antitumoral effect of H_4_R agonists in 1205Lu xenografted tumor induced in nude mice (**A**) Evaluation of relative tumor volume. Daily sc. treatment 1 mg/kg of histamine (HA), clozapine (CLZ) or JNJ28610244 (JNJ28) significantly diminished the tumor volume, evidencing this effect at the end of the experiment. Non-linear regression fit was performed to evaluate the exponential growth, (Repeated Measures ANOVA, ***P < 0.001 HA, CLZ and JNJ28 vs. control; Two-way ANOVA and Bonferroni post-test ^#^*P* < 0.05 JNJ28 vs. control). (**B**) 1205Lu tumors showed vertical growth (arrow), with invasion of reticular dermis (H&E staining, 200X-fold magnification). (**C**) 1205Lu tumors demonstrated intratumoral neutrophils (green arrow). (**D**) Dilated lymphatic vessels (red arrows, Masson´s trichromic staining, 200X-fold magnification). (**E**) Lymphatic emboli (arrow, H&E staining, 400X-fold magnification). Presence of tumor cells in mitosis. (**F**) HMB-45 positive and (**G**) Tyrosinase positive immunostaining. Scale bars = 100 and 20 μm. (**H**) Tumors of the control group showed significant anisocytosis and anisokaryosis, nuclear pleomorphism and atypical mitosis (yellow arrows). Tumors of mice treated with histamine, clozapine or JNJ28610244 presented cell homogeneity, with rounded and uniform nuclei with typical mitosis, and presence of inflammatory infiltrates (white arrows), (H&E staining, 400X-fold magnification). Immunohistochemistry of 1205Lu xenografted mice. Formalin-fixed paraffin embedded tissue sections of control, histamine, clozapine, and JNJ28610244 mice were stained to evaluate intracellular levels of histamine, HDC, H_4_R expression, proliferation and vascular and connective tissue morphology. Pictures were taken at a 400X-fold magnification for immunostaining (Scale bar = 20 μm) and 50X-fold magnification for trichromic stain (Scale bar = 100 μm).

Orthotopic xenografted tumors of the metastatic human melanoma cell line 1205Lu were developed into athymic nude mice. Figure 3A shows the results obtained from the tumor growth curves. Daily sc. treatment with 1 mg/kg of histamine, clozapine or JNJ28610244 for 30 days significantly diminished the tumor volume compared to those animals treated with saline solution (control group), (Figure 3A).

Given the aggressiveness of 1205Lu tumor model and following the standards and recommendations of animal care in cancer research, humanitarian death to those mice who presented one or more “endpoints” (e.g. ulceration / infection of the tumor site, weight loss > 20% body weight, etc.) was performed, making it impossible to perform the survival analysis that required to wait until the spontaneous death of the animals.

### Immunohistological and histochemical characteristics of 1205Lu xenografted tumors

Subcutaneous injection of 1205Lu cells in nude mice allowed the development of human melanoma tumors that exhibit a nodular growth pattern in the murine dermis, which resembles a melanoma in vertical growth phase frequently diagnosed in human patients (Figure 3B).

Histopathological analysis of H&E stained specimens revealed primary tumors characterized by fusiform cells in the periphery, with spindle nuclei without visible chromatin and intratumoral neutrophils presence in the control group. These tumors had epithelioid cells with marked anisocytosis and anisokaryosis, nuclear pleomorphism, presence of multinucleated giant cells and explosive mitosis (aberrant) (Figure 3C, 3H). Rich vascularization, invasion of deep dermis, ulceration, dilatation of lymph vessels and lymph emboli were also observed in untreated tumors (Figure 3D, 3E).

1205Lu tumors derived from treated animals showed a significant increase in lymphocytes infiltrate and poor intratumoral neutrophils presence, in contrast with tumors of the untreated animals (Figure 3H, Table 1, Supplementary Figure 2 of Supplementary Data).

**Table 1:** Histopathology and immunohistochemistry of 1205Lu xenografted tumor into nude mice

	Control	Histamine	Clozapine	JNJ28610244
**H_4_R^a^**	2.0 ± 0.6	3.5 ± 0.3	2.5 ± 0.3	3.5 ± 0.3
**Histamine ^a^HDC ^a^**	3.5 ± 0.93.5 ± 0.8	3.0 ± 0.62.0 ± 0.3	3.0 ± 0.62.0 ± 0.2	2.5 ± 0.31.0 ± 0.2*
**PCNA ^b^**	83.0 ± 9.0	35.0 ± 10.0*	40.0 ± 4.0*	24.0 ± 7.6***
**Mitotic index ^c^**	19.0 ± 2.0	9.0 ± 2.0*	12.0 ± 3.0*	10.0 ± 2.0*
**Vessels ^d^**	19.0 ± 1.0	12.0 ± 3.0*	12.0 ± 2.0*	10.0 ± 3.0*
**Neutrophils ^e^**	27.0 ± 9.8	7.0 ± 1.5	3.4 ± 0.9^#^	4.8 ± 2.0^#^
**Lymphocytes^f^**	5.0 ± 1.2	7.0 ± 1.9	11.0 ± 3.8	16.0 ± 3.8^#^

^a^ Score of stained cells; ^b^ Percentage of positive nuclei; ^c^ number of cells with visible chromosomes at 400X magnification in 5 random fields; ^d^ number of intratumoral vessels at 200X magnification in 10 random fields (hot spots); ^e^ number of neutrophils with visible segmented nucleus at 400X magnification in 5 random fields, ^f^ number of lymphocytes at 400X magnification in 5 random fields Results were expressed as mean ± SEM; *P < 0.05, **P < 0.01, ***P < 0.001 vs. Control group; Unpaired *t*-test. ^#^*P* < 0.05 *vs*. Control group; ANOVA Dunnett's Multiple Comparison post-Test.

Lineage specificity was corroborated in 1205Lu tumors by HMB-45 and TYR positive staining (Figure 3F, 3G). Immunohistochemical studies revealed that tumors of all groups exhibited significant levels of H_4_R and intracellular histamine. Non-significant differences in the expression levels of those antigens were observed between control and treated groups. However, tumors treated with the specific H_4_R agonist JNJ28610244, showed a significant reduction in the expression levels of histidine decarboxylase. Furthermore, proliferation of tumor cells evaluated by PCNA expression and MI were significantly diminished in tumors of histamine, clozapine and JNJ28610244 treated mice (Figure 2H), (Table 1).

In addition, the vascular morphology exhibited significant differences between tumor vessels of treated animals and those observed in the control group. Large and medium size vessels were predominant in intratumoral areas of control tumors, with vascular gaps and tortuous aberrant morphology, while capillaries and medium size vessels were observed in histamine, clozapine and JNJ28610244 treated tumors (Figure 2H). Intratumoral neovascularization showed a significant reduction in vessel number detected in histamine, clozapine and JNJ28610244 treated animals (*P* < 0.05, Table 1). Complementary microphotographs of each antigen are shown in Supplementary Figure 2A of Supplementary Data.

### Evaluation of metastatic potential

**Figure 4:**
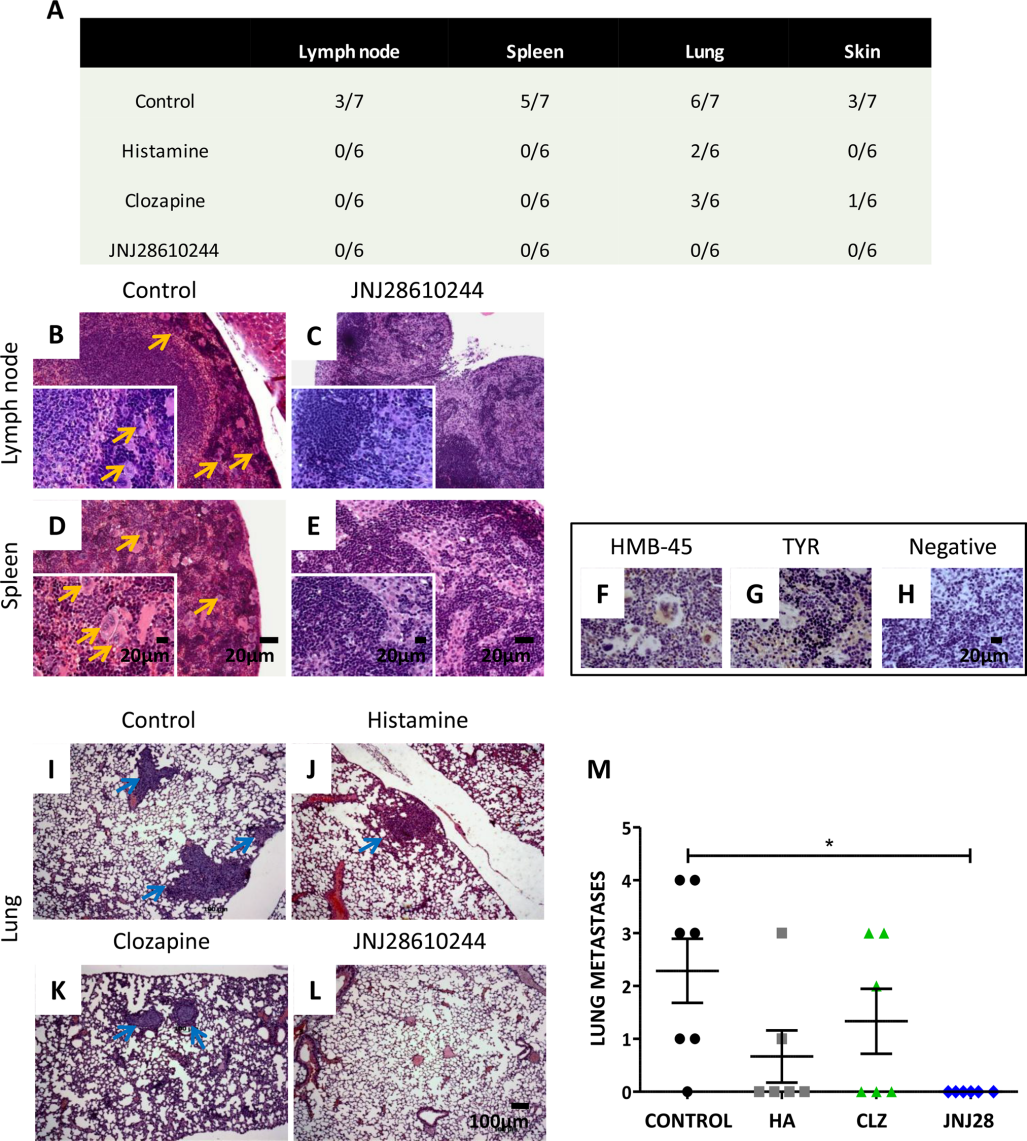
Analysis of the metastatic potential of 1205Lu xenografted tumor in mice (**A**) Number of animals with metastases of the total number of animals in the experimental group. (**B**) Giant multinucleated cells (yellow arrows) in lymph nodes and (**D**) in spleen of control mice. (**C**) Lymph nodes and (**E**) spleen of 1 mg/kg JNJ28610244 treated mice showing no particularities (H&E staining, 100X-fold magnification, inset: 400X-fold magnification). Scale bar = 20 μm. (**F**) Representative pictures of HMB-45 and (**G**) tyrosinase (TYR) positive immunostaining of giant multinucleated cells of spleen, confirming linage specificity. (**H**) Spleen of treated animals did not give signal for these antigens (negative immunostaining), confirming the absence of metastases. Pictures were taken at 400X-fold magnification. Scale bar = 20 μm. Representative images of lungs from (**I**) control, (**J**) histamine, (**K**) clozapine and (**L**) JNJ28610244 groups. Blue arrows indicate presence of lung metastases (H&E, 50X-fold magnification, scale bar = 100 μm). (**M**) The number of lung metastases was evaluated in mice treated with saline solution (control), 1 mg/kg histamine (HA), clozapine (CLZ,) or JNJ28610244 (JNJ28). Each dot represents the number of metastasis per mouse. The middle line represents the average number (Kruskal-Wallis non-parametric Test and Dunn's Multiple Comparison test, *P = 0.0214).

After studies on primary tumors, necropsy of all animals was performed in order to evaluate metastatic spread of 1205Lu cells to lymph nodes, spleen, lungs, skin, liver, heart, brain and bone marrow. Results are summarized in Figure 4A.

In control group, 43 % of the animals exhibited micrometastases on lymph nodes (including locoregional nodes) (Figure 4B), 71 % spleen metastases (Figure 4D), 85% lung metastases (Figure 4I) and 43 % showed metastases in the skin. Positive immunostaining of HMB-45 and TYR antigens was observed in multinucleated giant cells of lymph nodes and in giant cells of the spleen (Figure 4F, 4G), as well as in lung and skin metastases (data not shown), confirming the specificity of melanocytic lineage.

In contrast, animals treated with histamine showed occurrence of lung metastases only (33 %) (Figure 4J). Mice treated with clozapine exhibited lung (Figure 4K) and skin metastases (50 % and 17 %, respectively). All other organs examined were metastasis free.

Interestingly, no metastases in any of the tissue slides tested were detected in animals treated with JNJ28610244 (Figure 4A, 4C, 4E, 4L, 4M).

In line with these results, it is well established the involvement of extracellular matrix-degrading enzymes, such as matrix metalloproteinase MMP2, in melanoma progression and metastasis [27]. *In vitro* results demonstrate that H_4_R agonists (histamine, clozapine and VUF8430) decreased the gelatinolytic activity of MMP2 in 1205Lu cells (Figure 2E).

It is important to highlight that no adverse effects were found in the physical conditions of the experimental animals or in their behavior with the treatments employed. Moreover, non-toxic evidence was found under microscopic analysis inspection of the organs. Besides, none aberrant or side effects over the population elements of bone marrow or stromal alterations were observed (Supplementary Figure 3 of Supplementary Data).

### Expression levels of H_4_R, histidine decarboxylase and histamine in human benign melanocytic lesions (nevi) and in malignant melanomas. Correlation analysis between H_4_R levels and proliferation in human melanoma

**Figure 5:**
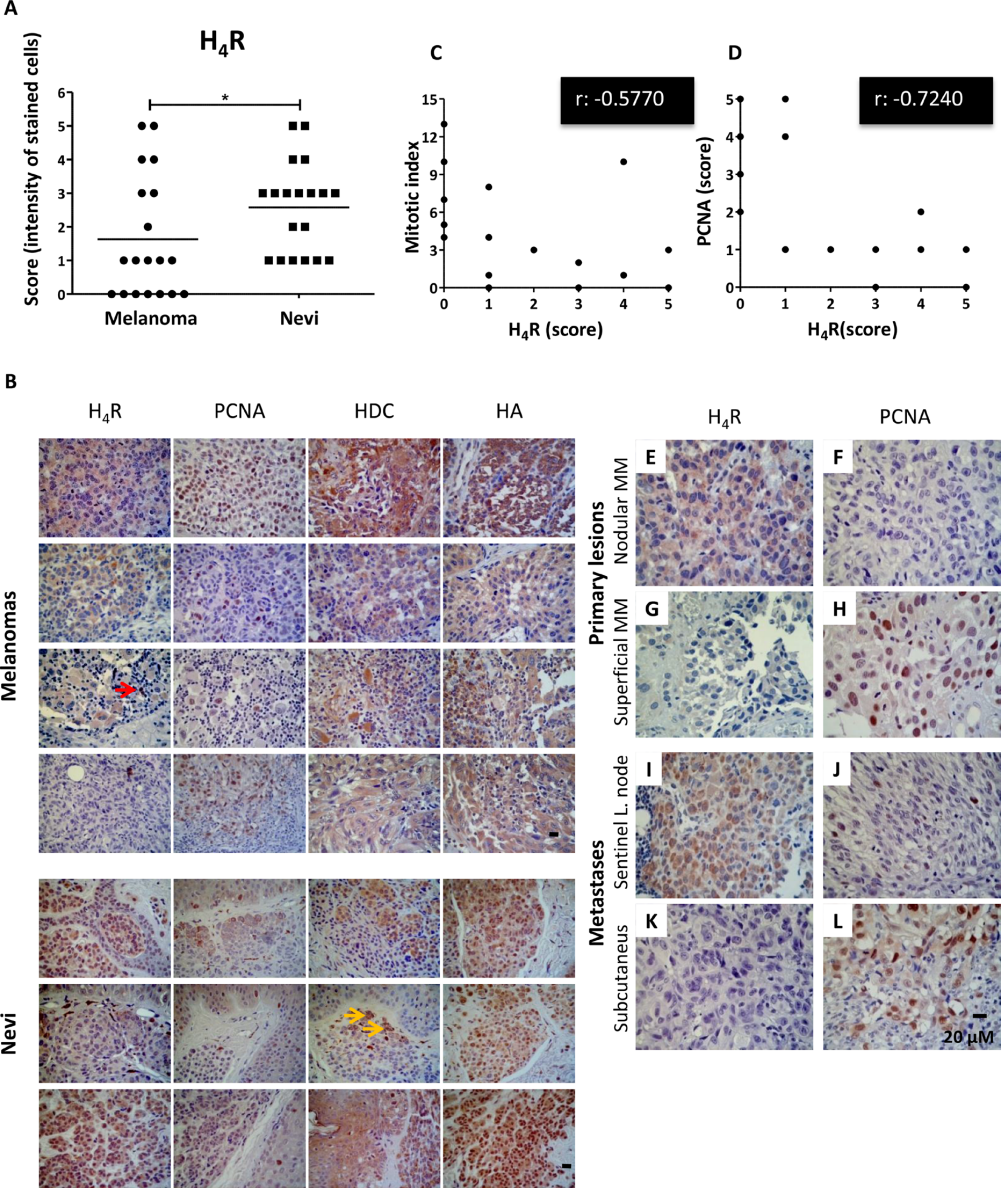
Immunohistochemical detection of H_4_R, histamine, HDC and PCNA in benign and malignant lesions derived from human melanocytic tissues (**A**) H_4_R expression level in nevi and melanoma tissues. Each dot represents a score (intensity of stained cells). The middle line represents the average number (Mann-Whitney non-parametric two tail Comparison Test, *P = 0.0452). (**B**) Representative pictures show examples of immunostaining for H_4_R, histamine, HDC and PCNA in superficial spreading melanoma, nodular melanoma, acral lentiginous amelanotic melanoma, subcutaneous melanoma metastasis, intradermal nevus, and junctional nevus. Red arrow indicates lymphoid cells positively stained. Yellow arrows indicate melanin content. Pictures were taken at 630X-fold magnification. Scale bar = 20 μm. (**C**) Spearman's inverse correlation between H_4_R and mitotic index (correlation coefficient rho, *r*: −0.5770, **P = 0.0097); (**D**) Spearman's inverse correlation between H_4_R and PCNA expression (correlation coefficient rho, *r*: −0.7240, ***P = 0.0005). Primary melanoma: (**E**, **F**) Nodular melanoma with high H_4_R expression levels and low PCNA; (**G**, **H**) Superficial amelanotic melanoma with low expression of H_4_R and high PCNA levels. Metastases: (**I**, **J**) Sentinel lymph node with melanoma partial metastases with high H_4_R levels and low PCNA expression, (**K**, **L**) Subcutaneous melanoma metastasis with low H_4_R expression and high PCNA levels. Pictures were taken at 630X-fold magnification. Scale bar = 20 mm.

Immunohistochemical analysis indicates that 100% (19/19) of benign biopsies and 63% (12/19) of melanomas were positive for the expression of H_4_R (Figure 5A, 5B), exhibiting lower expression levels of the receptor subtype in melanoma lesions (*P* = 0.0452). Histidine decarboxylase expression was detected in 89.5% (17/19) of malignant tissues and in 79% (15/19) of nevi. The enzyme levels were significantly higher in melanomas (*P* = 0.0096). Additionally, histamine intracellular content was detected in high levels, showing no significant differences between groups (*P* = 0.9639) (Figure 5B).

Analysis of PCNA expression and MI in the 19 specimens with diagnosis of malignant melanoma showed a direct correlation between them, consistent with previous studies that addressed this issue and postulated PCNA antigen as a prognostic marker of this disease [28, 29] (Spearman rho correlation coefficient r: 0.7706, ***P = 0.0001).

Interestingly, H_4_R expression levels inversely correlated with MI evaluated in melanoma biopsies (Figure 5C, Spearman rho correlation coefficient *r*: −0.5770, **P = 0.0097) and with PCNA expression (Figure 5B, 5D, Spearman rho correlation coefficient *r*: −0.7240, ***P = 0.0005), reinforcing the idea of an important relationship between the expression levels of this receptor subtype and the rate of proliferation exclusively in melanomas (Figure 5E–5L).

Correlation analysis performed in benign biopsies showed no significant results (data not shown). In addition, the analysis between histidine decarboxylase or intracellular histamine and proliferation markers (Supplementary Figure 2B of Supplementary Data) or against H_4_R expression levels in malignant tissue (data not shown) showed no significant correlations.

### Combined therapy using histamine and radiation in an experimental model of melanoma

Considering the complexity and poor prognosis of melanoma, new combination therapies need to be explored.

**Figure 6:**
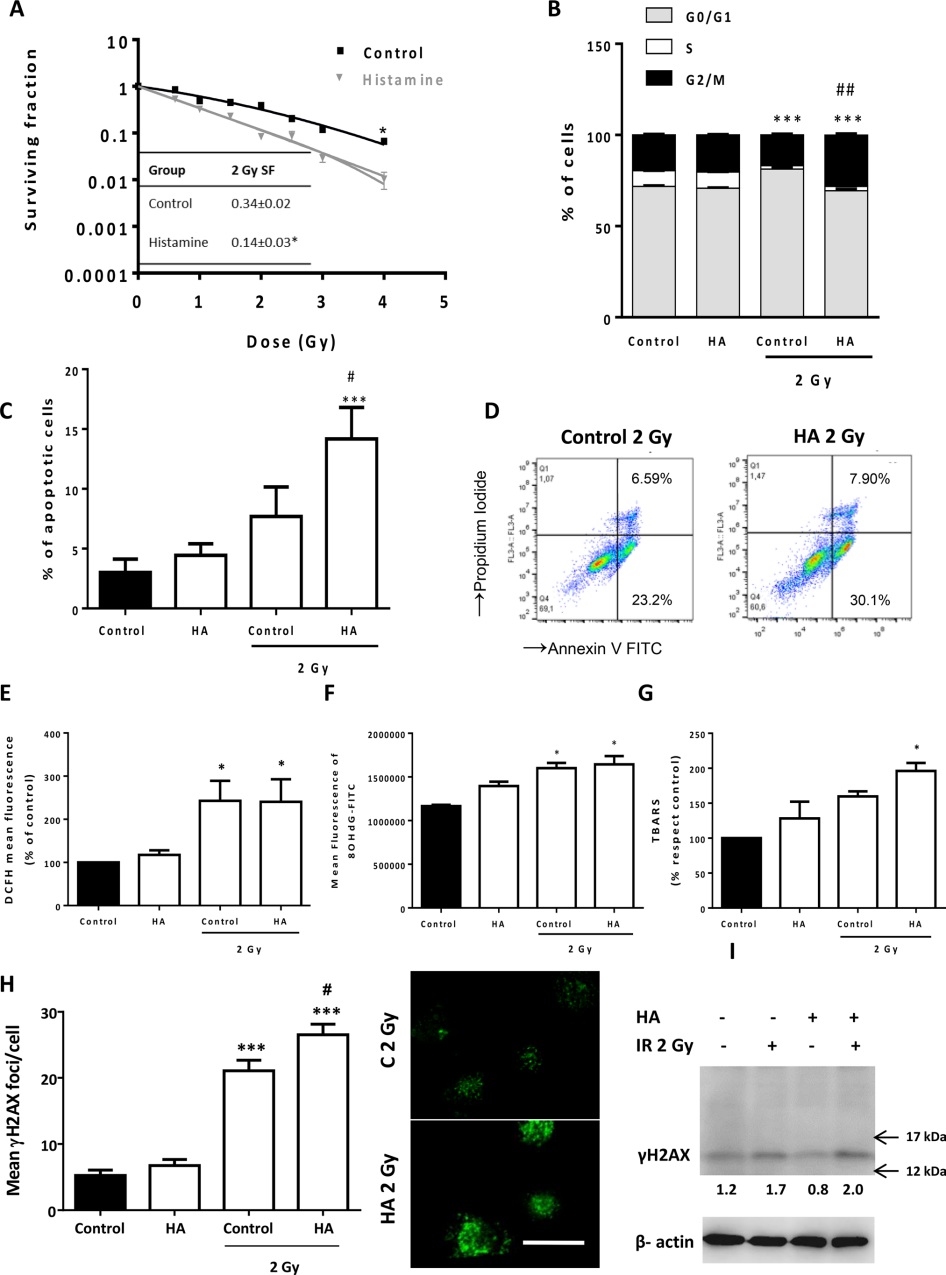
Effect of histamine on the radiosensitivity of melanoma cells 1205Lu cells were cultured in presence or absence of histamine (HA) and were irradiated 24 h after treatment. (**A**) Clonogenic survival was determined. Radiobiological parameters of 1205Lu cells were obtained from the survival curves adjusted to the linear quadratic model [SF= e^−(αD+βD2)^]. Values are means ± SEM of 3 independent experiments performed in triplicates. Inset: 2 Gy SF of untreated and histamine-treated cells. (**B**) The percentage of cells in different phases of the cell cycle was monitored 24 h after irradiation using flow cytometry. Results represent the mean value of 3 independent experiment (***P < 0.001 vs. % S phase of Control; ^##^*P* < 0.01 vs. % G2/M phase of 2 Gy Control. ANOVA and Newman-Keuls post test). (**C**) Percentage of apoptotic cells was determined 24 h after irradiation by the TUNEL assay (***P < 0.001 vs. Control; ^#^*P* < 0.05 vs. 2 Gy Control. ANOVA and Newman-Keuls post test), and by (**D**) Annexin-V staining and flow cytometry. Annexin-V positive cells are shown in both right quadrants of dot plot. (**E**) Measurement of intracellular ROS by flow cytometry (*P < 0.05, ANOVA and Newman-Keuls post test). (**F**) Mean fluorescence analysis of 8-OHdG determined by flow cytometry (*P < 0.05 vs. Control, ANOVA and Newman-Keuls post test). (**G**) Percentage of thiobarbituric acid reactive species (TBARS) with respect to untreated cells (control), (*P < 0.05 vs. Control, ANOVA and Newman-Keuls post test). (**H**) DNA double strand breaks were evidenced by γH2AX foci formation. The average number of foci per cell was determined 20 min after irradiation. Representative pictures were taken at a 400X-fold magnification (Scale bar = 20 μm). (**I**). γH2AX (15 kDa) was assayed by Western blot. β-actin (42 kDa) was used as loading control. Semiquantitative analyses of band intensities are shown (*n* = 3).

In an effort to improve outcomes even further, we next evaluated the anti-proliferative efficacy of the combined therapy of histamine with ionizing radiation. The combination demonstrated significant inhibition of cell proliferation and histamine radiosensitized 1205Lu cells (fraction of surviving cells after exposure to 2 Gy dose, 2 Gy SF: 0.14 ± 0.03 vs. 0.34 ± 0.02) (Figure 6A). The combination of histamine and radiation induced cell cycle accumulation in G2/M phase (Figure 6B) while significantly enhanced apoptosis in 1205Lu cells (Figure 6C, 6D). We have previously reported that histamine modulated oxidative stress and DNA damage in irradiated breast cancer cells. In 1205Lu melanoma cells, 2 Gy radiation dose increased ROS and 8-OHdG, a marker of oxidative DNA damage, in both histamine treated and non-treated cells (Figure 6E, 6F). Nevertheless, histamine potentiated radiation-induced double strand breaks, enhancing γH2AX foci (Figure 6H, 6I) and lipid peroxidation as evidenced by an increase in TBARS levels (Figure 6G). Non-significant changes in the radiobiological parameter were observed with two H_4_R agonists, suggesting that other histamine receptor is involved in histamine radiosensitization. In this regard, an H_3_R agonist mimicked histamine effect when combined with radiation (Supplementary Figure 4A, 4B of Supplementary Data).

**Figure 7:**
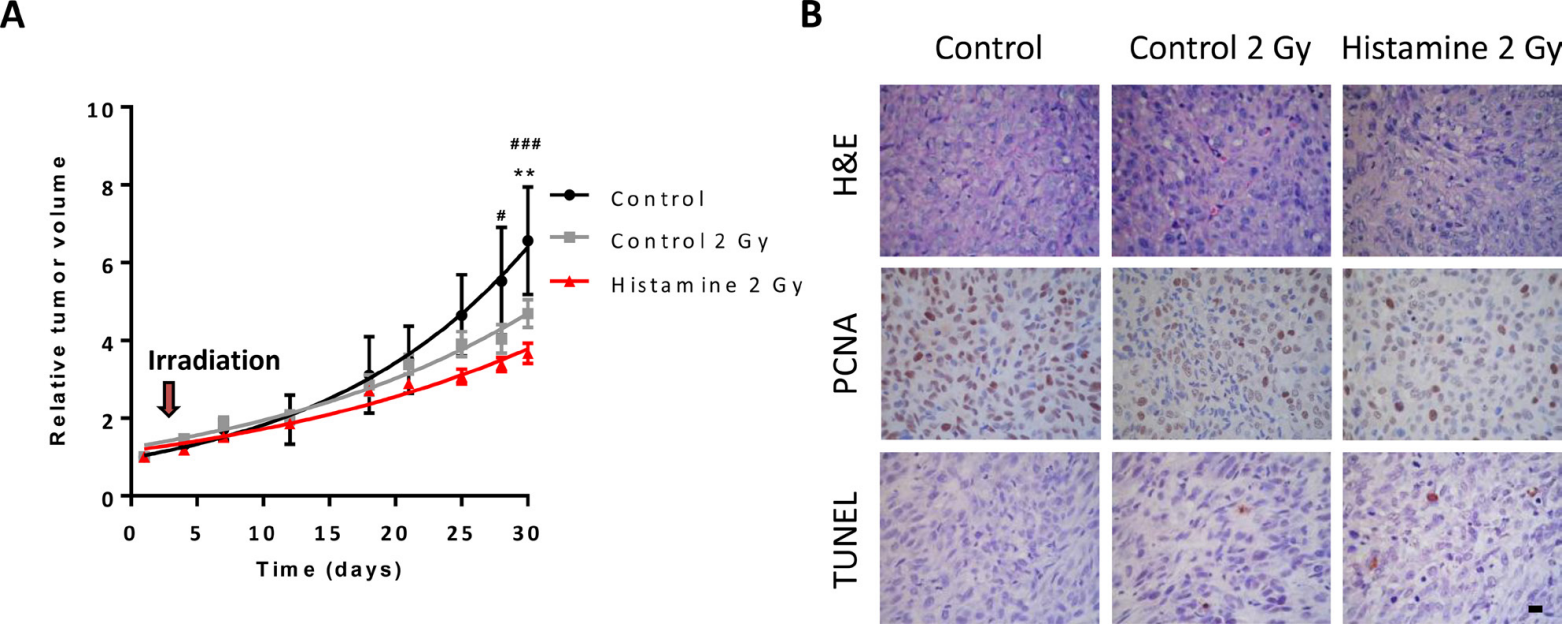
Combined effect of radiation and histamine on melanoma tumors induced in nude mice (**A**) Evaluation of relative tumor volume in untreated (control), untreated and 2 Gy irradiated (control 2 Gy) and histamine-treated and 2 Gy irradiated animals (Histamine 2 Gy). Tumor volumes were measured by day and non-linear regression fit was performed to evaluate the exponential growth, (Repeated Measures ANOVA and Dunnet post-test, *P < 0.01 Histamine 2 Gy vs. control; Two-way ANOVA and Bonferroni post-test ^#^*P* < 0.05, ^###^*P* < 0.001 Histamine 2 Gy vs. control). (**B**) Histological and immunohistochemical analysis. Formalin-fixed paraffin embedded tissue sections of the different groups were stained to evaluate histopathological characteristics (H&E), proliferation (PCNA) and apoptosis (TUNEL). 630X original magnification. Scale bar = 20 μm.

Nude mice with established subcutaneous 1205Lu tumors were employed to evaluate the effect of the combination therapy *in vivo*. Histamine treatment (1 mg/kg. day, sc. administration) potentiated the antitumoral effect of radiation (5 doses of 2 Gy), decreasing tumor size and increasing tumor doubling time (Figure 7A, Table 2). Histopathological analysis demonstrated that radiation increased the fibrous connective tissue while decreased the PCNA proliferation marker. Histamine further decreased proliferation marker expression (Figure 7B). Apoptosis was rare and a slight increase was observed in histamine treated and irradiated tumors (Figure 7B).

**Table 2:** Effect of radiation and histamine treatment on tumor doubling time

	Control	Histamine	Control 2 Gy	Histamine 2 Gy
**Tumor doubling time (days)**	11.2 ± 1.0	14.7 ± 1.0	17.1 ± 1.1	21.1 ± 5.7*

Tumor doubling time of each group is depicted numerically. Results were expressed as mean ± SEM, ANOVA and Newman-Keuls Multiple Comparison post Test, (*P < 0.05 vs. Control).

## DISCUSSION

The H_4_R discovery with functional presence in a wide range of tissues, including some tumors, revealed new features of histamine and new perspectives in the pharmacology of this receptor [30–32]. In order to strengthen the observations obtained in a model of primary melanoma developed with human M1/15 melanoma cells [9] and further study the role of H_4_R in malignant progression, evaluating the effect of treatments against tumor invasion and spread, a metastatic melanoma model was investigated using the invasive 1205Lu cells [19, 20].

Among agonists, the atypical neuroleptic clozapine has been demonstrated to activate the H_4_R, as it was previously reported [9, 31, 33]. To further investigate some functional characteristics of the H_4_R, a more selective H_4_R agonist, JNJ28610244 compound, was employed *in vitro* and *in vivo*. This experimental compound has demonstrated excellent potency and selectivity for the H_4_R and thus, serves as a useful pharmacological tool for exploring and better understanding the role of H_4_R [34].

*In vitro* studies showed that 1205Lu cells express H_4_R at mRNA and protein levels. Treatment with histamine, clozapine or JNJ28610244 significantly reduces proliferation, which was associated with an increase in cell senescence and an induction of melanogenesis and extensions development, main characteristics of terminal melanocytic differentiation. In agreement with these results, histamine and H_4_R agonists decreased the proliferation and induced apoptosis and cell senescence of human breast cancer cells [33, 35] while suppressed human cholangiocarcinoma progression, decreasing tumor invasion and growth [36].

*In vivo* studies show that histamine, clozapine or JNJ28610244 reduce tumor growth after 4 weeks of treatment. Accordingly, these compounds significantly decreased tumor volume *in vivo* in a human triple negative breast cancer experimental model [33]. Furthermore, the H_4_R agonist 4-methylhistamine significantly decreased tumor volume and increased survival of mice bearing xenograft non-small cell lung cancer tumors [37].

*Ex vivo* studies revealed that 1205Lu tumors exhibit a nodular growth pattern in mice dermis like M1/15 melanoma experimental model [9]. Histologically, 1205Lu tumors were highly undifferentiated with presence of multiple giant cells, explosive mitosis, with a rich vasculature, neutrophils infiltrate, presenting dermis invasion and rapid ulceration. Balch et al., demonstrated that ulcerated lesions exhibit minimal inflammatory response, which worsen the prognosis and decrease patient survival from 80% to 55% compared to non-ulcerated melanomas, in which Breslow index is significant [38]. In contrast, tumors from treated animals are characterized by a predominance of epithelioid cells with typical mitosis and an increased lymphocytes and decreased neutrophils infiltrates. The immune system is involved in the pathogenesis of cancer, and may contribute either to disease progression or to inhibit tumor growth. A prominent lymphocyte infiltration and dendritic cells are considered as a favorable prognostic factor in malignant melanoma and metastatic melanoma, increasing the survival of patients [39, 40]. Clinical data of pigmented lesions have shown that the absence of tumor lymphocytic infiltration, increased intratumoral neutrophils, high Breslow thickness, presence of ulceration and masculine sex, predict the increased occurrence of lymph node metastases in patients with primary cutaneous melanoma undergoing sentinel node biopsy [41, 42, 43]. Cancer models developed in athymic nude mouse, widely used to study human tumors, have impaired immune system and thus, it is not possible to effectively evaluate the role of the immunity in tumor progression and response to anti-cancer treatments. Further studies in melanoma syngeneic models with normal host immune system are needed to identify the role of tumor immunity and characterize the tumor-infiltrating lymphocytes and other immune cells in histamine´s effect on melanoma.

Immunohistochemical studies show significant levels of H_4_R, but non-significant changes upon treatments were observed. Interestingly, treatment with the selective H_4_R agonist JNJ28610244 produces a significant reduction of tumoral histidine decarboxylase expression levels, enzyme that has been found elevated in several tumors [5]. In addition, MI and tumoral PCNA expression are diminished in animals treated with histamine, clozapine or JNJ28610244. MI is an important prognostic factor, predicting overall survival and response to chemotherapy. Mitotic rate was considered the strongest prognostic factor after tumor thickness in primary melanoma [44]. Additionally, PCNA correlates directly with patient survival and metastatic tumor behavior [45, 46].

Lymphogenous and hematogenous metastasis can occur in melanoma [47]. In this sense, H_4_R agonists together with a reduction of tumor volume, produce changes in the morphology and the number of vessels. Both findings are in accordance with previous results obtained in M1/15 primary melanoma model [9]. The molecular mechanisms underlying the effects of these compounds on angiogenesis remain unknown and deserve further investigation. Numerous studies show that histamine is involved in the angiogenesis and might exert pro- or anti-angiogenic effects, depending on the concentration employed, the presence of co-factors and/or the tumor microenvironment characteristics [48–50].

Interestingly, histological analysis revealed dilated lymph vessels, often containing tumor emboli, in tumors of the control group. These intrinsic characteristics of invasive and metastatic tumors are not observed in animals treated with histamine, clozapine or JNJ28610244.

Cutaneous melanoma is considered to have a high metastatic potential. Control animals showed melanoma metastasis in lymph nodes, spleen, skin and lungs. The lung is one of the most common sites of initial recurrence of this disease, accounting for 36–42% of initial metastases [51]. The number of animals affected with lung metastases decrease with histamine and clozapine while metastasis in spleen and lymph nodes are completely prevented with both treatments. Interestingly, JNJ28610244 treatment inhibits the occurrence of metastasis in all organs tested. These results support the hypothesis that primary tumors, which exhibit low levels of proliferation (PCNA and MI) and a reduced angiogenesis, are associated with decreased metastatic spread.

Metastasis is a multistep process, which includes proliferation, neovascularization, immune system evasion, lymphangiogenesis, invasion, circulation, embolism, extravasation and colonization [47]. Therefore, we consider that all these processes are potential mechanisms that could be involved in the reduction of metastatic spread along with results obtained in *in vitro* experiments, in which histamine and H_4_R agonists reduce cell proliferation, increase tumor cell senescence and differentiation and interestingly decrease the gelatinolytic activity of MMP-2, a regulator of tissue invasion.

In accordance with our previous published data [9, 33], no side toxic effects in organs were observed, indicating that these compounds could be administered safely at the concentration employed.

The presence of H_4_R in benign and malignant lesions of melanocytic lineage further highlights the potential clinical use of histamine for the treatment of melanoma. H_4_R expression levels were significantly higher in benign lesions than in malignant tissues. These results are in accordance with recently reported studies of colorectal adenomas and carcinomas biopsies, in which lower levels of H_4_R expression were detected in tumor tissues compared to cells of normal colonic mucosa [52, 53]. Furthermore, it was reported reduced expression of this receptor in advanced gastric cancer samples compared to normal tissue [54]. On the other hand, in cholangiocarcinoma, H_4_R expression levels are increased relative to non-malignant tissues [36]. Melanoma exhibits high histamine content [55]. In this context, results indicate that although histidine decarboxylase levels are significantly higher in melanomas than in nevi, and it is expressed in a greater number of malignant respect to benign lesions, the content of intracellular histamine was similar in both tissues. This observed effect may be due to differences in the histamine catabolism and/or in histidine decarboxylase activity between nevi and melanomas.

Regarding proliferation, PCNA expression levels and MI are significantly elevated in melanoma compared to benign biopsies. Interestingly, our studies revealed that H_4_R expression level in this tumor type is negatively correlated with both proliferation and prognostic markers. This fact demonstrates the association between low proliferation rate in melanomas that exhibit significant levels of this histamine receptor subtype. The study of a higher number of samples and the monitoring of patients is required to better understand the H_4_R role in human melanoma progression and to determine if it might be a prognostic marker for this disease.

Novel targeted therapies for metastatic melanoma were rationally design, like BRAF inhibitors and modulators of the immune response that interfere with T-cell function [2]. However, survival of melanoma patients is still disappointing and treatments such as monotherapy have significant limitations. Therefore, there is growing interest in using these therapeutic modalities in combination. The combined use of two targeted therapy drugs can treat advanced melanoma more effectively; however, it has more side effects. Despite melanoma has conventionally been considered as a radioresistant cancer that lead to limited indications for radiation [56], combined radiotherapy and targeted therapies or immunotherapy has led to resurgence of interest in radiation therapy for this disease [57].

In this regard, results further indicate that histamine enhances the response against ionizing radiation of 1205Lu cells. The rationale behind this combination is based on the potential radiosensitization of breast cancer cells with histamine previously reported [58].

In the present study, we show that histamine increases radiosensitivy of melanoma cells *in vitro*, inducing G2/M cell cycle arrest that is considered the most radiosensitive cell cycle phase [59]. In addition, histamine treatment enhances radiation-induced apoptosis, lipid peroxidation and DNA damage of melanoma cells. The radiosensitizing effect of histamine was further confirmed *in vivo*, where histamine improves radiation-induced decrease in tumor growth. These results agree with previous data in a breast cancer model in which histamine potentiates the anti-tumoral effects of radiation [58].

In the light of the above described findings, we conclude that H_4_R has a crucial role in the regulation of proliferation and progression of human melanoma. Stimulation of H_4_R by specific ligands may represent a novel therapeutic strategy in those tumors that expresses this receptor. Further studies and clinical trials will be needed to confirm the therapeutic potential of these compounds.

Finally, histamine may be used in combination therapy to increase efficacy and decrease toxicity for melanoma treatment, especially considering that it is being used in clinical trials as an adjuvant to immunotherapy.

## MATERIALS AND METHODS

### Chemicals

Histamine (HA), (Sigma Chemical Co., Missouri, USA); H_4_R agonists: VUF 8430 (VUF), (Tocris Bioscience, Ellisville, Missouri, USA); clozapine (CLZ), (kindly provided by Fabra Laboratories S.A, Buenos Aires, Argentina); JNJ28610244 (JNJ28), (Janssen Research & Development, San Diego, USA). H_3_R agonist: (R)(−)-α-Methylhistamine dihydrochloride (RαMeH), (Sigma Chemical Co., MO, USA). H_4_R antagonist: JNJ7777120 (JNJ77), (Janssen Research & Development, San Diego, USA).

### Cell culture

Human metastatic melanoma cell line, 1205Lu (ATCC^®^ CRL 2812^™^), was cultured in RPMI 1640 supplemented with 10% (v/v) FBS, 0.3 g/L glutamine, and 0.04 g/L gentamicin (all from Gibco BRL, Grand Island, NY, USA). Cells were maintained at 37°C in a humidified atmosphere containing 5% CO_2_.

### RT-PCR

The retrotranscription reaction was performed with 2 μg of RNA that was isolated using TRIZOL reagent, according to the manufacturer´s instructions (Invitrogen, USA). Negative controls were performed with water instead of cDNA. WM35 (American Type Tissue Culture Collection, USA), human primary melanoma cell line, was used as positive control to compare the expression of histamine H_4_ receptor [8]. Detection of histamine receptors and tyrosinase were carried out as it was described previously [8]. H_4_R fragment identity was corroborated by sequenciation (Macrogen, Korea). M1/15 melanoma cell line was used as positive control of tyrosinase expression. β-actin was used as load control. All primers and PCR conditions used are summarized in Supplementary Table 1 of Supplementary Data.

PCR products were subjected to gel electrophoresis and photographed using a Sony Cyber-Shot DSC-S75 camera.

### Western blot analysis

Cells were placed on ice and washed twice with cold phosphate-buffered saline (PBS). Cells were then scraped into a lysis buffer (100 mM Tris/HCl buffer, pH 8, containing 1% Triton X-100 and protease inhibitors) and incubated for 15 min on ice. After centrifugation at 6000 rpm for 10 min, the supernatants were used for protein determination according to Bradford assay [60].

Equal amounts of proteins (100 μg) were fractionated on SDS-polyacrylamide gels (12%) and transferred electrophoretically onto nitrocellulose membranes (Sigma Chemical Co., USA). Membranes were blocked and probed overnight with primary rabbit anti-H_4_R (1:500, catalog number H4R41-A, Alpha Diagnostic International, USA). WM35 melanoma cells were used as positive control and HEK293 human embryonic kidney cells as negative control to compare the expression of H_4_R.

For phosphorylated H2AX detection, rabbit anti-γH2AX (1:500, catalog number 2577 γH2AX, Cell Signaling Technology) was used.

Immunoreactivity was detected by using horseradish peroxidase-conjugated anti-rabbit IgG (catalog number A0545), (1:1000), (Sigma Chemical Co., MO, USA) and visualized by enhanced chemiluminescence (Amersham Biosciences, USA). Densitometric analyses were performed using the software ImageJ 1.32j version (NIH, Massachusetts, USA).

### Immunostaining

Cells were seeded into 12-well plates with coverslips in culture medium (25000 cells/well) for 48 h. The cells were washed twice with PBS and fixed for 15 min in 4% (v/v) formaldehyde in PBS. The cells were then incubated overnight in a humidified chamber at 4°C with primary rabbit anti-H_4_R (1:100, catalog number H4R41-A, Alpha Diagnostic International, USA). After washing, cells were incubated with FITC-conjugated anti-rabbit (1:400) (Bio-Rad Laboratories, USA), and nuclei were counterstained with ethidium bromide (Sigma Chemical Co., USA) at room temperature. Coverslips were mounted with FluorSaveTM Reagent (Calbiochem, USA) and fluorescence was evaluated by confocal microscopy using an LSM 510 Laser scanning microscope (Carl Zeiss). All photographs were taken at 400X or 1000X magnification using a Cool Snap digital camera and ZEN 2009 Light Edition Software (Carl Zeiss MicroImaging). WM35 and M1/15 melanoma cells were used as positive control. HEK293 human embryonic kidney cells were used as negative control to compare the expression of H_4_R.

### Cell proliferation assays

For clonogenic assay, 1205Lu cells were seeded in six-well plates (1000 cells/well). Cells were untreated (control) or treated with 0.01 to 10 μM of histamine, clozapine (diluted in 0.5% ethanol final concentration), JNJ28610244 and/or 10 μM JNJ7777120. Cells were incubated for 7 days and then fixed with 10% (v/v) formaldehyde in PBS (Sigma Chemical Co., Missouri, USA) and stained with 1% (w/v) toluidine blue in 70% (v/v) ethanol. The clonogenic proliferation was evaluated by counting the colonies containing 50 cells or more and was expressed as a percentage of the untreated wells.

Quantification of cellular DNA synthesis was performed by BrdU (Sigma Chemical Co., Missouri, USA) incorporation as previously described [9]. Cells were seeded into 12-well plates in culture medium (25000 cells/well), and treated with 10 μM of histamine, clozapine, JNJ28610244 and/or 10 μM of JNJ7777120 for 48 h. Light microscopy was performed on an Axiolab Carl Zeiss microscope (Göttingen, Germany). All photographs were taken at 630X magnification using a Canon Power-Shot G5 camera (Tokyo, Japan).

### Senescence-associated β-galactosidase staining

Cells were seeded into 12-well plates in culture medium (25000 cells/well) and were left untreated or treated with 10 μM histamine, clozapine, JNJ28610244 and/or 10 μM JNJ7777120 for 48 h. Senescence-associated β-galactosidase-positive cells were detected using the method described by Dimri et al. [61]. Briefly, cells were fixed and incubated at 37°C for 8 h with 1 mg/ml 5-bromo-4-chloro-indolyl-β-galactoside (USB Corp., USA) in an appropriate buffer. After incubation, cells were washed twice with PBS and counterstained with hematoxylin and the percentage of β-galactosidase-positive cells was assessed under light microscopy (Axiolab Carl Zeiss, Göttingen, Germany). All photographs were taken at 630X magnification using a Canon PowerShot G5 camera (Tokyo, Japan).

### L-dopa staining

Staining with L-dopa was performed using the method described by Hamoen et al. [62]. Cells were seeded into 12-well plates in culture medium (780 cells/well) and were untreated (control) or treated for 10 days with 10 μM histamine, clozapine, JNJ28610244 and/or 10 μM JNJ7777120. TPA (12-O-Tetradecanoylphorbol 13-acetate) was used as positive control of pigment formation and morphological changes in a final concentration of 16 nM [63, 64]. The location of dopa oxidase (tyrosinase) was indicated by the presence of an insoluble brown/black precipitate. All photographs were taken at 200X magnification using a Canon PowerShot G5 camera (Tokyo, Japan).

### Gelatin zymography

After 24 h in serum free RPMI medium, supernatants from cell cultures were collected, mixed with non-reducing buffer and electrophoresed on 7% SDS-polyacrylamide gels with 0.1% gelatin (Sigma, Chemical Co., MO, USA). The gels were washed with 0.5% Triton X-100 (v/v) in Tris-buffered saline (TBS), pH 7.4 for 30 min, rinsed briefly with TBS, and incubated in TBS, pH 7.4 supplemented with 1 mM Ca2+ at 37°C for 24 h. Gelatinolytic activity was visualized by staining zymograms with Coomassie Brilliant Blue G250 (Sigma, Chemical Co., MO, USA) and destaining in acetic acid-methanol-H_2_O (1:3:6). MCF-7 cell line was used as positive control of MMP2 activity. Activity of lytic bands was determined by densitometry employing the ImageJ 1.32j version (NIH, Massachusetts, USA) software. Results were expressed for each treatment as % of optical density respect to control cells (% MMP-2 activity).

### Treatments and animals

Histamine was diluted in saline solution. Clozapine and JNJ28610244 were diluted in 0.1 N HCl, neutralized with 4 N NaOH and finally diluted with saline solution.

Specific pathogen-free athymic male nude (NIH nu/nu) mice were purchased from the Division of Laboratory Animal Production, School of Veterinary Sciences, University of La Plata, Buenos Aires (Argentina), and maintained in sterile isolated conditions. Mice were kept 5–10 per cage and maintained in our animal health care facility at 22 to 24°C and 50% to 60% humidity on a 12 h light/dark cycle with food and water available *ad libitum*. Animals with an age of 8–10 weeks and an average weight of 25 g were used. All animal protocols were supervised and managed by qualified trained personnel according to international guidelines for animal care.

The animal procedures were in accordance with recommendations from the National Institute of Health Guide for the Care and Use of Laboratory Animals (NIH Publications No. 8023) and the Guidelines for the welfare and use of animals in cancer research [65, 66, 67]. All procedures involving animals were reviewed and approved by the Ethical Committee for the Use and Care of Laboratory Animals of the School of Pharmacy and Biochemistry.

1205Lu cells (2.5 × 10^6^) were collected by centrifugation and resuspended in 100 μL RPMI-1640 (GIBCO, Grand Island, New York, USA). Human melanoma tumors were induced by subcutaneous (sc) injection of human melanoma cells into the right flank of twenty-five male athymic nude mice. The animals were randomly separated according to Johnson and Besselsen [68], into four groups and received a subcutaneous daily injection of saline solution (control group, *n* = 7), 1 mg/kg histamine (HA, *n* = 6), 1 mg/kg clozapine (CLZ, *n* = 6) or 1 mg/kg JNJ28610244 (JNJ28, *n* = 6).

### Tumor growth

The length and width of the subcutaneous tumors were measured using a caliper three times a week. The tumor size was calculated as sphere volume according to the following formula: Tumor volume [cm^3^] = 4/3π × r [cm]^3^. The tumor growth curve was constructed with relative tumor volume (tumor volume measured with respect to initial tumor volume at the beginning of treatment) and analysis was carried out using GraphPad Prism version 6. The exponential growth equation was Y_t_ =Y_0_xe^(kxt)^, where Y_0_ was the initial tumor volume that increased exponentially with a rate constant, k. The tumor doubling time was calculated as 0.69/k.

Treatments lasted 30 days, the animals were sacrificed by cervical dislocation at the end of the experiment or when they presented one or more criteria of death (endpoints) [66, 69].

### Histochemistry and immunostaining

Tumors were excised, fixed in 4% (v/v) formaldehyde in PBS (formalin), paraffin embedded and sliced into 3–4 μm thick sections to evaluate the expression levels of H_4_R (1:75, Catalog number H4R41-A, Alpha Diagnostic International, TX, USA), HDC (1:100, Catalog number B-GP 265-1, Euro-Diagnostica, Sweden) and histamine (1:100, catalog number H7403, Sigma Chemical Co., MO, USA).

Cell growth was assessed by determining the PCNA (1:100, clone PC10, Dako Cytomation, Denmark) expression and by mitotic index (MI), as the number of cells with visible chromosomes in 400X magnification fields. Melanocytic linage was evaluated by expression of TYR (1:75, clone T311) and HMB-45 (1:75, clone HMB45, Dako Cytomation, Denmark). The appropriate secondary HRP-conjugated antiserum was employed in each case. 3,3-diaminobenzidine (DAB) tablets were used for staining and hematoxylin for counterstaining.

Histopathological examination was performed on hematoxylin-eosin (H&E) and Masson´s trichromic stained specimens (Biopur diagnostic, Buenos Aires, Argentina). Vascular morphology was evaluated on stained sections at 50X magnification to identify the largest vascular areas around the tumor. In these hot spots, intratumoral vascularity was evaluated by counting vessels at 200X magnification in 10 random fields. Number of neutrophils with visible segmented nucleus and number of lymphocytes were evaluated on H&E stained sections by counting cells at 400X magnification in 5 random fields.

For H_4_R, histidine decarboxylase and histamine level evaluation, staining diffuse positive cytoplasms (and/or perinuclear staining for TYR and HMB-45) were considered as positive cells. For the content of H_4_R, histamine and histidine decarboxylase, a score based on the intensity of uniform color was determined as: 0 (not detectable), 1 (very low), 2 (low), 3 (moderate), 4 (high), 5 (very high). PCNA percentage was evaluated by counting positive nuclei on the total number of nuclei in 15 random fields. These scorings were previously published [70–72].

Metastatic spread analysis was performed over cuts of excised organs. They were fixed in 4% (v/v) formaldehyde in PBS, embedded in paraffin and cut into sections of 3–4 microns. Tissue morphology was examined on tissue sections after H&E staining and immunodetection of H_4_R, histamine, histidine decarboxylase, PCNA and TYR and HMB-45 was performed as described above.

An overall examination of staining was carried out at 10X magnification, and representative area of specimen was then viewed at 630X magnification. Visualization was performed with an optical microscope (Axiolab Carl Zeiss, Germany). The photographs were taken at 50X, 100X, 200X, 400X or 630X, as appropriate, with Canon Power Shot G5 camera (Japan). To control the signal specificity, serial sections were made from two selected positive cases, which were subjected to the same staining procedure with, either a normal rabbit IgG or PBS to replace the first antibody. This control staining did not give rise to a signal. As positive controls, tissue sections known by literature expressing these antigens were used. The immunostaining assessment was performed blinded to the clinical data by consensus agreement of two observers (Massari N, Herrero Ducloux V).

### Toxicity analysis

Treated mice were examined daily to assess overall fitness and observe the appearance of potential adverse or toxic effects. Priority was given to the observation of sudden changes in weight, decreased appetite, skin disorders, pruritus, motor involvement, appearance of blood in urine and/or feces, diarrhea, excessive salivation and/or changes in behavior until the animals were killed or died spontaneously.

Furthermore, to perform histopathological observations, several organs (skin, lymph nodes, spleen, liver, kidneys, heart, lungs, and brain) were surgically removed and fixed with 4% (v/v) formaldehyde in PBS. Bone marrows were removed, fixed in Bouin's solution and also studied. All tissues were embedded in paraffin and sectioned at 3–4 μm. Histologic sections were evaluated using H&E staining.

### Human melanocytic tissues biopsies

Tissue samples were obtained from paraffin blocks belonging to historic reserve material (kindly provided and selected from tumor bank of Pathology Department, José María Penna Hospital, Buenos Aires). The study was in accordance to the latest version of the Declaration of Helsinki and was approved by the Ethic Committee of the School of Pharmacy and Biochemistry, University of Buenos Aires.

19 surgically obtained tissues with diagnosis of malignant melanoma from patients who received no pretreatment (superficial spreading melanoma, *n* = 4; acral lentiginous melanoma, *n* = 1; nodular melanoma, *n* = 9; metastases from melanoma, *n* = 5), and 19 benign melanocytic lesions (intradermal nevus, *n* = 13; junctional nevus, *n* = 3; compound nevus, *n* = 3) were used. The characteristics of the malignant biopsies are summarized in Supplementary Table 2 of Supplementary Data.

Tissue morphology, mitotic index (MI) and the immunodetection of H_4_R, histamine, histidine decarboxylase or PCNA were performed as previously described.

### Experiments using gamma radiation

### Radiation dose-response curves

For the radiosensitivity studies, 1205Lu cells were seeded in 6-well plates and were treated with 10 μM of histamine, JNJ28610244, VUF8430, (R)(−)-α-Methylhistamine or remained untreated. The radiobiological parameter 2Gy SF (fraction of surviving cells after exposure to 2 Gy dose) of 1205Lu cells was obtained from the survival curves adjusted to the linear quadratic model [SF= e^−(αD+βD2)^]), as it was previously described [73].

### Cell cycle analysis

Cells were plated into 6-well plates (100000 cells/well), cultured for 24 h and serum-starved for an additional 24 h. Treatments were added to synchronized cell cultures 24 h before irradiation with a single dose of 2 Gy, and were maintained up to 24 h after. Cells were harvested by trypsinazation, fixed with ice-cold methanol and stained with propidium iodide (PI) staining solution (50 μg/mL in PBS containing 0.2 mg/mL of DNase-free RNase A; Sigma Chemical Co., MO, USA). Cell cycle distribution was evaluated by flow cytometry and data analysis was performed using BD AccuriCSampler software (Becton Dickinson Co.).

### Determination of apoptosis

Cells were seeded into 12-well plates in culture medium (25000 cells/well) and remained untreated (control) or treated with 10 μM histamine and irradiated with a 2 Gy dose or not 24 h later, and maintained up to 24 h when determination of apoptosis was performed. Apoptotic cells were determined by TdT-mediated UTP-biotin Nick End labeling (TUNEL) (CHEMICON International, CA, USA) assay and by staining with Annexin-V FITC (BD Biosciences, USA) using flow cytometry, both according to the manufacturer's instructions.

### Measurement of intracellular ROS production

Cells untreated (control) or treated with 10 μM histamine and irradiated or not with a 2 Gy dose were incubated with 5 μM dichlorodihydrofluorescein diacetate (DCFH2-DA) (Sigma Chemical Co., MO, USA) for 30 min at 37°C. Cells were then washed, detached by trypsinazation, and suspended in PBS. Levels of intracellular ROS were measured immediately by flow cytometry and data analysis was performed using BD AccuriCSampler software (Becton Dickinson Co.).

### Evaluation of TBARS levels

The thiobarbituric acid reactive species (TBARS) assay is used to monitoring lipid peroxidation. The method used in the present study, was described by Yagi et al. [73], and adapted as previously reported [74]. A molar extinction coefficient of ε = 1.56 × 10^5^/M/cm was used for calculations.

### Determination of 8-OHdG by flow cytometry

Cells untreated (control) or treated with 10 μM histamine for 24 h, and irradiated or not with a 2 Gy dose were then washed, detached by trypsinazation, fixed with methanol at −20°C. Fixed cells were treated with RNase (100 μg/ml) for 1 h at 37°C and proteinase K (10 μg/ml) (Sigma Chemical Co.) for 10 min at room temperature. DNA was denatured and after blocking, cells were incubated 30 min at room temperature with goat 8-OHdG (1:100, catalog number AB5830, Millipore, Temecula, CA, USA). Cells were washed with PBS and incubated for 30 min with 1:300 FITC-conjugated anti-goat IgG and mean fluorescence was determined by flow cytometry and data analysis was performed using BD AccuriCSampler software (Becton Dickinson Co.).

### Immunofluorescent γH2AX staining

Cells were seeded on coverslips in 12-well plates (25000 cells/well) and allowed to grow overnight. Cells remained untreated or were treated with 10 μM histamine and irradiated 24 h after with a single dose of 2 Gy. Cells were washed and fixed with 4% (v/v) paraformaldehyde 20 min after irradiation. After blocking in 10% normal blocking serum at room temperature for 10 min, slides were incubated with rabbit anti-phosphorilated histone H2AX antibody (γH2AX, 1:100, catalog number 2577, Cell Signaling Technology, Beverly, MA) at 4°C overnight and then incubated with goat anti-rabbit IgG conjugated with FITC and Dapi at room temperature (Sigma Chemical Co., MO, USA). Coverslips were mounted and visualized as we described above. For quantification of foci, a minimum of 100 cells were analyzed.

### Animals and treatments

For experiments using radiation, xenografted mice were separated into 4 groups when tumor volumes reached 8 mm in diameter, making possible localized irradiation. The untreated group received a sc. daily injection of saline solution (control, *n* = 5), histamine group received a sc daily injection of histamine 1 mg/kg (treated, *n* = 5), untreated 2 Gy group received a sc. daily injection of saline solution (untreated and irradiated animals, *n* = 5) or histamine 2 Gy group received a sc. daily injection of histamine 1 mg/kg (treated and irradiated, *n* = 5). One day after treatment started, animals were irradiated with a 2 Gy dose per day, for 5 consecutive days. Mice were anesthetized with a combination of xylazine (10 mg/kg) and ketamine (100 mg/kg) and fixed on an acryl plate. Xenografts were locally irradiated with a ^60^Co γ-radiation source (Teradi 800; Hospital Municipal de Oncología “Marie Curie”), while other body parts were protected with lead blocks [66].

The length and width of the tumors were measured and the tumor size was calculated as we described above. Animals were euthanized by cervical dislocation at the end of the experiment (30 days) or when they presented one or more criteria of death (endpoints) [66, 69], to perform the *ex vivo* histological studies.

### Statistical analysis

Representative results are presented as means +/− standard error of the mean (SEM). Statistical evaluations were made by Unpaired *t*-Test, analysis of variance (ANOVA) that was followed by Dunnet test, Newman-Keuls Multiple Comparison Test, Bonferroni Test. Kruskal-Wallis non-parametric test, followed by Dunn's Multiple Comparison test was used to compare average number of metastases and Mann-Whitney non-parametric test was used to compare average scores of staining intensity or percentages. For determination of the association among H_4_R, PCNA expression and MI, Spearman's rho correlation coefficients and two- tailed significances were determined. All statistical analyses were performed with GraphPad Prism version 6.00^™^ (CA, USA).

## SUPPLEMENTARY MATERIALS FIGURES AND TABLES



## Abbreviations

ANOVA: analysis of variance
BrdU: 5-bromo-20-deoxyuridine
cDNA: complementary deoxyribonucleic acid
CLZ: clozapine
DAB: 3,3-diaminobenzidine
Dapi: 4′-6-diamidino-2-phenylindole
DCFH-DA: dichlorodihydrofluorescein diacetate
DNA: deoxyribonucleic acid
ERK1/2: extracellular signal–regulated kinases
FITC: fluorescein isothiocyanate
Gy: gray
H&E: hematoxylin-eosin
HA: histamine
HDC: histidine decarboxylase
HMB-45: human melanoma black 45
HxR: histamine Hx receptor
IgG: immunoglobuline G
IL: interleukin
JNJ28: JNJ28610244
JNJ77: JNJ7777120
MAPK: mitogen-activated protein kinase
MI: mitotic index
MM: malignant melanoma
mRNA: messenger ribonucleic acid
NADPH: nicotinamide adenine dinucleotide phosphate
NK: natural killer
PBS: phosphate buffered saline
PCNA: proliferating cell nuclear antigen
PVDF: poly-(vinylidine difluoride)
ROS: reactive oxygen species
RαMeH: (R)(−)-α-Methylhistamine dihydrochloride
RT-PCR: reverse transcription polymerase chain reaction
SF: surviving fraction
SDS: sodium dodecyl sulfate
TPA: 12-O-Tetradecanoylphorbol 13-acetate
TUNEL: terminal deoxynucleotidyl transferase dUTP nick end labeling
TYR: tyrosinase
VEGF: Vascular endothelial growth factor
μM: micromolar
8-OHdG: 8-hydroxy-20-deoxyguanosine
γH2AX: phosphorylated histone H2AX.

## References

[1] Block KI, Gyllenhaal C, Lowe L, Amedei A, Amin AR, Amin A, Aquilano K, Arbiser J, Arreola A, Arzumanyan A, Ashraf SS, Azmi AS, Benencia F (2015). Designing a broad-spectrum integrative approach for cancer prevention and treatment. Semin Cancer Biol.

[2] Marzuka A, Huang L, Theodosakis N, Bosenberg M (2015). Melanoma Treatments: Advances and Mechanisms. J Cell Physiol.

[3] Bernatchez C, Cooper ZA, Wargo JA, Hwu P, Lizée G (2016). Novel Treatments in Development for Melanoma. Cancer Treat Res.

[4] Prieto PA1, Reuben A, Cooper ZA, Wargo JA (2016). Targeted therapies combined with immune checkpoint therapy. Cancer J.

[5] Medina VA, Rivera ES (2010). Histamine receptors and cancer pharmacology. Br J Pharmacol.

[6] Gutzmer R, Gschwandtner M, Rossbach K, Mommert S, Werfel T, Kietzmann M, Baeumer W.A (2011). Pathogenetic and therapeutic implications of the histamine H4 receptor in inflammatory skin diseases and pruritus. Front Biosci.

[7] De Benedetto A, Yoshida T, Fridy S, Park JE, Kuo IH, Beck LA (2015). Histamine and Skin Barrier: Are Histamine Antagonists Useful for the Prevention or Treatment of Atopic Dermatitis?. J Clin Med.

[8] Massari NA, Medina VA, Martinel Lamas DJ, Cricco GP, Croci M, Sambuco L, Bergoc RM, Rivera ES (2011). Role of H4 receptor in histamine-mediated responses in human melanoma. Melanoma Res.

[9] Massari NA, Medina VA, Cricco GP, Martinel Lamas DJ, Sambuco L, Pagotto R, Ventura C, Ciraolo PJ, Pignataro O, Bergoc RM, Rivera ES (2013). Antitumor activity of histamine and clozapine in a mouse experimental model of human melanoma. J Dermatol Sci.

[10] Medina VA, Coruzzi G, Martinel Lamas DJ, Massari N, Adami M, Levi-Schaffer F, Ben-Zimra M, Schwelberger H, Rivera ES, Stark H. (2013). Histamine in cancer. Chapter 8 in: Histamine H4 receptor: A novel drug target in immunoregulatory and inflammatory diseases. Versita Warsaw/Poland.

[11] Hegyesi H, Somlai B, Varga VL, Toth G, Kovacs P, Molnar EL, Laszlo V, Karpati S, Rivera E, Falus A, Darvas Z (2001). Suppression of melanoma cell proliferation by histidine decarboxylase specific antisense oligonucleotides. J Invest Dermatol.

[12] Pós Z, Sáfrány G, Müller K, Tóth S, Falus A, Hegyesi H (2005). Phenotypic profiling of engineered mouse melanomas with manipulated histamine production identifies histamine H2 receptor and rho-C as histamine-regulated melanoma progression markers. Cancer Res.

[13] Pos Z, Wiener Z, Pocza P, Racz M, Toth S, Darvas Z, Molnar V, Hegyesi H, Falus A (2008). Histamine suppresses fibulin-5 and insulin-like growth factor-II receptor expression in melanoma. Cancer Res.

[14] Agarwala SS, Glaspy J, O'Day SJ, Mitchell M, Gutheil J, Whitman E, Gonzalez R, Hersh E, Feun L, Belt R, Meyskens F, Hellstrand K, Wood D (2002). Results from a randomized phase III study comparing combined treatment with histamine dihydrochloride plus interleukin-2 versus interleukin-2 alone in patients with metastatic melanoma. J Clin Oncol.

[15] Hellstrand K, Brune M, Naredi P, Mellqvist UH, Hansson M, Gehlsen KR, Hermodsson S (2000). Histamine: a novel approach to cancer immunotherapy. Cancer Invest.

[16] Hegyesi H, Horváth B, Pállinger E, Pós Z, Molnár V, Falus A (2005). Histamine elevates the expression of Ets-1, a protooncogen in human melanoma cell lines through H2 receptor. FEBS Lett.

[17] Engkilde K, Thyssen JP, Menné T, Johansen JD (2011). Association between cancer and contact allergy: a linkage study. BMJ Open.

[18] Hajdarbegovic E1, Atiq N, van der Leest R, Thio B, Nijsten T (2014). Atopic dermatitis is not a protective factor for melanoma but asthma may be. Int J Clin Oncol.

[19] Juhasz I, Albelda SM, Elder DE, Murphy GF, Adachi K, Herlyn D, Valyi-Nagy IT, Herlyn M (1993). Growth and invasion of human melanomas in human skin grafted to immunodeficient mice. Am J Pathol.

[20] Graeven U, Herlyn M (1992). *In vitro* growth patterns of normal human melanocytes and melanocytes from different stages of melanoma progression. J Immunother.

[21] Nguyen T, Shapiro DA, George SR, Setola V, Lee DK, Cheng R, Rauser L, Lee SP, Lynch KR, Roth BL, O'Dowd BF (2001). Discovery of a novel member of the histamine receptor family. Mol Pharmacol.

[22] Lippert U, Artuc M, Grützkau A, Babina M, Guhl S, Haase I, Blaschke V, Zachmann K, Knosalla M, Middel P, Krüger-Krasagakis S, Henz BM (2004). Human skin mast cells express H2 and H4, but not H3 receptors. J Invest Dermatol.

[23] van Rijn RM, van Marle A, Chazot PL, Langemeijer E, Qin Y, Shenton FC, Lim HD, Zuiderveld OP, Sansuk K, Dy M, Smit MJ, Tensen CP, Bakker RA (2008). Cloning and characterization of dominant negative splice variants of the human histamine H4 receptor. Biochem J.

[24] Atwood BK, Lopez J, Wager-Miller J, Mackie K, Straiker A (2011). Expression of G protein-coupled receptors and related proteins in HEK293, AtT20, BV2, and N18 cell lines as revealed by microarray analysis. BMC Genomics.

[25] Hearing VJ, Jiménez M (1989). Analysis of mammalian pigmentation at the molecular level. Pigment Cell Res.

[26] Potterf SB, Hearing VJ (1998). Tyrosine transport into melanosomes is increased following stimulation of melanocyte differentiation. Biochem Biophys Res Commun.

[27] Gould Rothberg BE, Bracken MB, Rimm DL (2009). Tissue biomarkers for prognosis in cutaneous melanoma: a systematic review and meta-analysis. J. Natl Cancer Inst.

[28] Ben-Izhak O, Bar-Chana M, Sussman L, Dobiner V, Sandbank J, Cagnano M, Cohen H, Sabo E (2002). Ki67 antigen and PCNA proliferation markers predict survival in anorectal malignant melanoma. Histopathology.

[29] Cianfrocca M, Goldstein LJ (2004). Prognostic and predictive factors in early-stage breast cancer. Oncologist.

[30] Lim HD, Adami M, Guaita E, Werfel T, Smits RA, de Esch IJ, Bakker RA, Gutzmer R, Coruzzi G, Leurs R (2009). Pharmacological characterization of the new histamine H4 receptor agonist VUF 8430. Br J Pharmacol.

[31] Leurs R, Chazot PL, Shenton FC, Lim HD, de Esch IJ (2009). Molecular and biochemical pharmacology of the histamine H4 receptor. Br J Pharmacol.

[32] Kiss R, Keserű GM (2012). Histamine H4 receptor ligands and their potential therapeutic applications: an update. Expert Opin Ther Pat.

[33] Martinel Lamas DJ, Croci M, Carabajal E, Crescenti EJ, Sambuco L, Massari NA, Bergoc RM, Rivera ES, Medina VA (2013). Therapeutic potential of histamine H4 receptor agonists in triple-negative human breast cancer experimental model. Br J Pharmacol.

[34] Yu F, Wolin RL, Wei J, Desai JP, McGovern PM, Dunford PJ, Karlsson L, Thurmond RL (2010). Pharmacological characterization of oxime agonists of the histamine H4 receptor. J Receptor Ligand Channel Res.

[35] Medina VA, Brenzoni PG, Lamas DJ, Massari N, Mondillo C, Nunez MA, Pignataro O, Rivera ES (2011). Role of histamine H4 receptor in breast cancer cell proliferation. Front Biosci (Elite Ed).

[36] Meng F, Han Y, Staloch D, Francis T, Stokes A, Francis H (2011). The H4 histamine receptor agonist, clobenpropit, suppresses human cholangiocarcinoma progression by disruption of epithelial mesenchymal transition and tumor metastasis. Hepatology.

[37] Cai WK, Hu J, Li T, Meng JR, Ma X, Yin SJ, Zhao CH, He GH, Xu GL (2014). Activation of histamine H4 receptors decreases epithelial-to-mesenchymal transition progress by inhibiting transforming growth factor-β1 signalling pathway in non-small cell lung cancer. Eur J Cancer.

[38] Balch CM, Gershenwald JE, Soong SJ, Thompson JF, Atkins MB, Byrd DR, Buzaid AC, Cochran AJ, Coit DG, Ding S, Eggermont AM, Flaherty KT, Gimotty PA (2009). Final version of 2009 AJCC melanoma staging and classification. J Clin Oncol.

[39] Fitzpatrick TB (2009). Dermatología En Medicina General.

[40] Ladányi A (2013). Prognostic value of tumor-infiltrating immune cells in melanoma. Magy Onkol.

[41] Taylor RC, Patel A, Panageas KS, Busam KJ, Brady MS (2007). Tumor-infiltrating lymphocytes predict sentinel lymph node positivity in patients with cutaneous melanoma. J Clin Oncol.

[42] Jensen TO, Schmidt H, Møller HJ, Donskov F, Høyer M, Sjoegren P, Christensen IJ, Steiniche T (2012). Intratumoral neutrophils and plasmacytoid dendritic cells indicate poor prognosis and are associated with pSTAT3 expression in AJCC stage I/II melanoma. Cancer.

[43] Ladányi A (2015). Prognostic and predictive significance of immune cells infiltrating cutaneous melanoma. Pigment Cell Melanoma Res.

[44] Balch CM, Soong SJ, Gershenwald JE, Thompson JF, Coit DG, Atkins MB, Ding S, Cochran AJ, Eggermont AM, Flaherty KT, Gimotty PA, Johnson TM, Kirkwood JM (2013). Age as a prognostic factor in patients with localized melanoma and regional metastases. Ann Surg Oncol.

[45] Goldmann T, Ribbert D, Suter L, Brode M, Otto F (1998). Tumor characteristics involved in the metastatic behaviour as an improvement in primary cutaneous melanoma prognostics. J Exp Clin Cancer Res.

[46] Niezabitowski A, Czajecki K, Ryś J, Kruczak A, Gruchała A, Wasilewska A, Lackowska B, Sokołowski A, Szklarski W (1999). Prognostic evaluation of cutaneous malignant melanoma: a clinicopathologic and immunohistochemical study. J Surg Oncol.

[47] Adler NR, Haydon A, McLean CA, Kelly JW, Mar VJ (2017). Metastatic pathways in patients with cutaneous melanoma. Pigment Cell Melanoma Res.

[48] Ohtsu H, Watanabe T (2003). New functions of histamine found in histidine decarboxylase gene knockout mice. Biochem Biophys Res Commun.

[49] Ghosh AK, Hirasawa N, Ohuchi K (2001). Enhancement by histamine of vascular endothelial growth factor production in granulation tissue via H receptors. Br J Pharmacol.

[50] Francis H, DeMorrow S, Venter J, Onori P, White M, Gaudio E, Francis T, Greene JF, Tran S, Meininger CJ, Alpini G (2012). Inhibition of histidine decarboxylase ablates the autocrine tumorigenic effects of histamine in human cholangiocarcinoma. Gut.

[51] Lee JH, Gulec SA, Kyshtoobayeva A, Sim MS, Morton DL (2009). Biological factors, tumor growth kinetics, and survival after metastasectomy for pulmonary melanoma. Ann Surg Oncol.

[52] Boer K, Helinger E, Helinger A, Pocza P, Pos Z, Demeter P, Baranyai Z, Dede K, Darvas Z, Falus A (2008). Decreased expression of histamine H1 and H4 receptors suggests disturbance of local regulation in human colorectal tumours by histamine. Eur J Cell Biol.

[53] Fang Z, Yao W, Xiong Y, Li J, Liu L, Shi L, Zhang W, Zhang C, Nie L, Wan J (2011). Attenuated expression of HRH4 in colorectal carcinomas: a potential influence on tumor growth and progression. BMC Cancer.

[54] Zhang C, Xiong Y, Li J, Yang Y, Liu L, Wang W, Wang L, Li M, Fang Z (2012). Deletion and down-regulation of HRH4 gene in gastric carcinomas: a potential correlation with tumor progression. PLoS One.

[55] Pós Z, Hegyesi H, Rivera E, Falus A (2004). Histamine and cell differentiation. Histamine: Biology and Medical Aspects.

[56] Khan MK, Khan N, Almasan A, Macklis R (2011). Future of radiation therapy for malignant melanoma in an era of newer, more effective biological agents. Onco Targets Ther.

[57] Fort M, Guet S, Husheng S, Calitchi E, Belkacemi Y, AROME (Association of Radiotherapy & Oncology of the Mediterranean arEa; www.aromecancer.org), TRONE (Transatlantic Radiation Oncology NEtwork) (2016). Role of radiation therapy in melanomas: Systematic review and best practice in 2016. Crit Rev Oncol Hematol.

[58] Martinel Lamas DJ, Cortina JE, Ventura C, Sterle HA, Valli E, Balestrasse KB, Blanco H, Cremaschi GA, Rivera ES, Medina VA (2015). Enhancement of ionizing radiation response by histamine *in vitro* and *in vivo* in human breast cancer. Cancer Biol Ther.

[59] Hall EJ, Giaccia AJ, Hall EJ, Giaccia AJ (2012). Radiobiology for Radiologists.

[60] Bradford MM (1976). A rapid and sensitive method for the quantitation of microgram quantities of protein utilizing the principle of protein-dye binding. Anal Biochem.

[61] Dimri GP, Lee X, Basile G, Acosta M, Scott G, Roskelley C, Medrano EE, Linskens M, Rubelj I, Pereira-Smith O (1995). A biomarker that identifies senescent human cells in culture and in aging skin *in vivo*. Proc Natl Acad Sci USA.

[62] Hamoen KE, Borel Rinkes IH, Morgan JR (2001). Hepatocyte growth factor and melanoma: gene transfer studies in human melanocytes. Melanoma Res.

[63] Ziegler-Heitbrock HW, Munker R, Johnson J, Petersmann I, Schmoeckel C, Riethmüller G (1985). *In vitro* differentiation of human melanoma cells analyzed with monoclonal antibodies. Cancer Res.

[64] Kiguchi K, Collart FR, Henning-Chubb C, Huberman E (1990). Induction of cell differentiation in melanoma cells by inhibitors of IMP dehydrogenase: altered patterns of IMP dehydrogenase expression and activity. Cell Growth Differ.

[65] Bayne K (1996). Revised Guide for the Care and Use of Laboratory Animals available American Physiological Society. Physiologist.

[66] Workman P, Aboagye EO, Balkwill F, Balmain A, Bruder G, Chaplin DJ, Double JA, Everitt J, Farningham DA, Glennie MJ, Kelland LR, Robinson V, Stratford IJ (2010). Guidelines for the welfare and use of animals in cancer research. Br J Cancer.

[67] Guide for the Care and Use of Laboratory Animals (2011). National Research Council (US) Committee for the Update of the Guide for the Care and Use of Laboratory Animals.

[68] Johnson PD, Besselsen DG (2002). Practical aspects of experimental design in animal research. ILAR J.

[69] Guidelines for Endpoints in Animal Study Proposals (2013). http://oacu.od.nih.gov/ARAC/index.htm/.

[70] Blancato J, Singh B, Liu A, Liao DJ, Dickson RB (2004). Correlation of amplification and overexpression of the c-myc oncogene in high-grade breast cancer: FISH, *in situ* hybridisation and immunohistochemical analyses. Br J Cancer.

[71] Erbil Y, Oztezcan S, Giriş M, Barbaros U, Olgaç V, Bilge H, Küçücük H, Toker G (2005). The effect of glutamine on radiation-induced organ damage. Life Sci.

[72] Medina V, Cricco G, Nuñez M, Martín G, Mohamad N, Correa-Fiz F, Sanchez-Jimenez F, Bergoc R, Rivera ES (2006). Histamine-mediated signaling processes in human malignant mammary cells. Cancer Biol Ther.

[73] Yagi K (1976). A simple fluorometric assay for lipoperoxide in blood plasma. Biochem Med.

[74] Martinel Lamas DJ, Carabajal E, Prestifilippo JP, Rossi L, Elverdin JC, Merani S, Bergoc RM, Rivera ES, Medina VA (2013). Protection of radiation-induced damage to the hematopoietic system, small intestine and salivary glands in rats by JNJ7777120 compound, a histamine H4 ligand. PLoS One.

[75] Visús C, Andres R, Mayordomo JI, Martinez-Lorenzo MJ, Murillo L, Sáez-Gutiérrez B, Diestre C, Marcos I, Astier P, Godino J, Carapeto-Marquez de Prado FJ, Larrad L, Tres A (2007). Prognostic role of circulating melanoma cells detected by reverse transcriptase-polymerase chain reaction for tyrosinase mRNA in patients with melanoma. Melanoma Res.

