# Supplementary material for: Histamine therapeutic efficacy in metastatic melanoma: Role of histamine H4 receptor agonists and opportunity for combination with radiation
Source: Oncotarget. 2017 Feb 21;8(16):26471–91. doi: 10.18632/oncotarget.15594 (PMC5432273; doi:10.18632/oncotarget.15594)
Supplement: Supplementary file 1 [file oncotarget-08-26471-s001.pdf]

Histamine therapeutic efficacy in metastatic melanoma: Role of histamine H_4 receptor agonists and opportunity for combination with radiation

Supplementary Materials

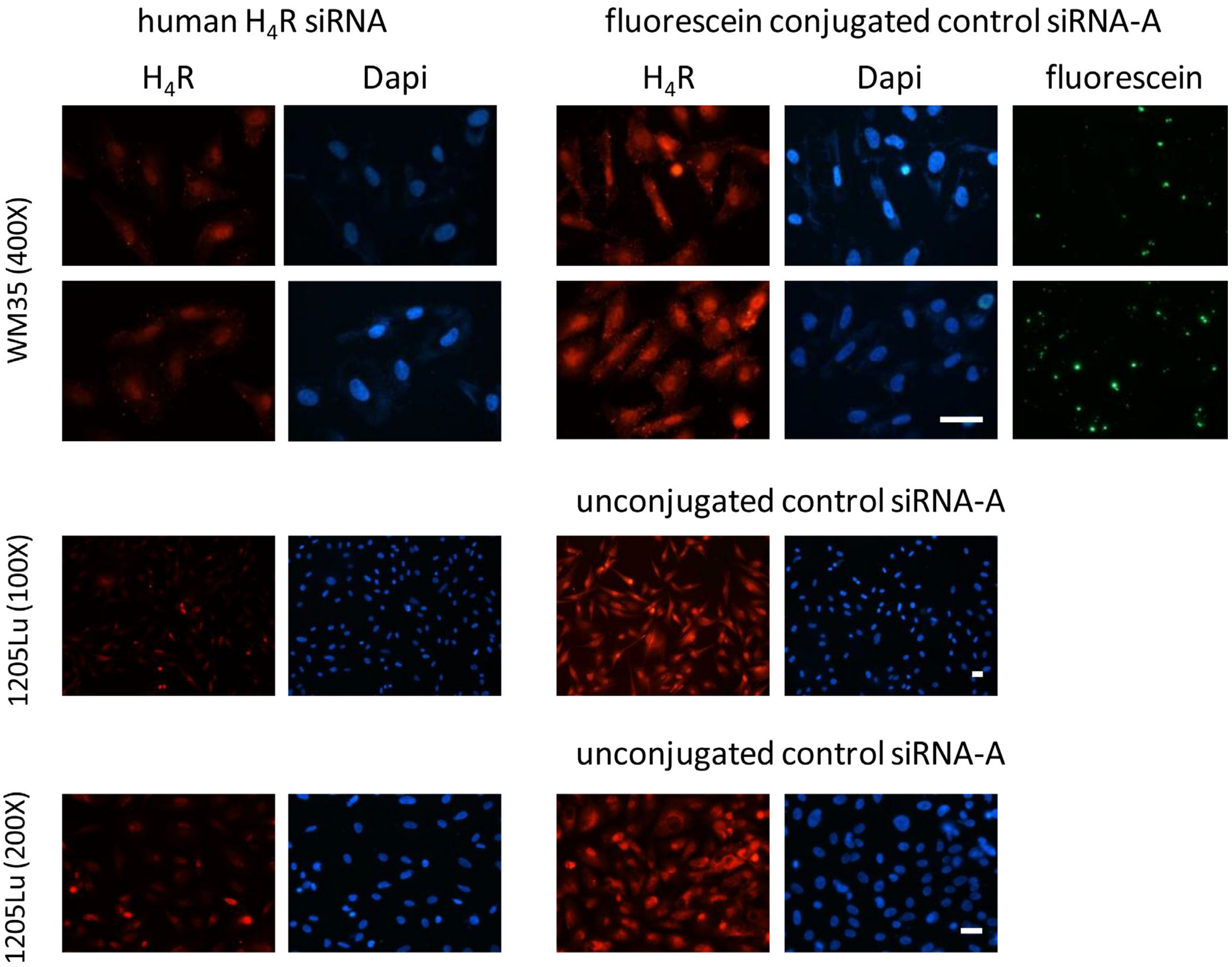

Supplementary Figure 1: Small interfering RNA (siRNA) H_4R silencing. The specificity of H_4R antibody was evaluated by immunofluorescence in transfected cells. siRNA specific for H_4R mRNA was used to knock down its expression in melanoma cells. Transfection optimization was performed by the evaluation of the H_4R protein and mRNA, as it was described in Massari et al., 2011. H_4R siRNA: cells transfected with specific sequences siRNA designed to knockdown H_4R gene expression. Fluorescein conjugated control siRNA-A or unconjugated control siRNA-A (negative control of transfection): cells transfected with scrambled siRNA. WM35 and 1205Lu melanoma cells were transfected according to the manufacturer's instructions using Lipofectamine 2000 (Invitrogen), 8 µl (80 pmol) of human H_4R siRNA (sc-40025) pools of three to five target-specific 19–25 nucleotides siRNAs designed to knockdown H_4R gene expression, 8 µl (80 pmol) of scrambled fluorescein conjugated control siRNA-A (sc-36869) or scrambled unconjugated control siRNA-A (sc-37007), both negative controls that consists of scrambled sequences that will not lead to the specific degradation of any cellular message (shared no homology to the human genome), all from Santa Cruz Biotechnology, Santa Cruz, CA, USA. Transfection was performed during 6 h. Then cells were washed with PBS and fresh medium was added. Decreased expression of H_4R was evaluated by immunocytochemistry analysis 18 h post-transfection Pictures were taken at 400X, 200X or 100X, scale bar = 20 µM. Two different fields are shown.

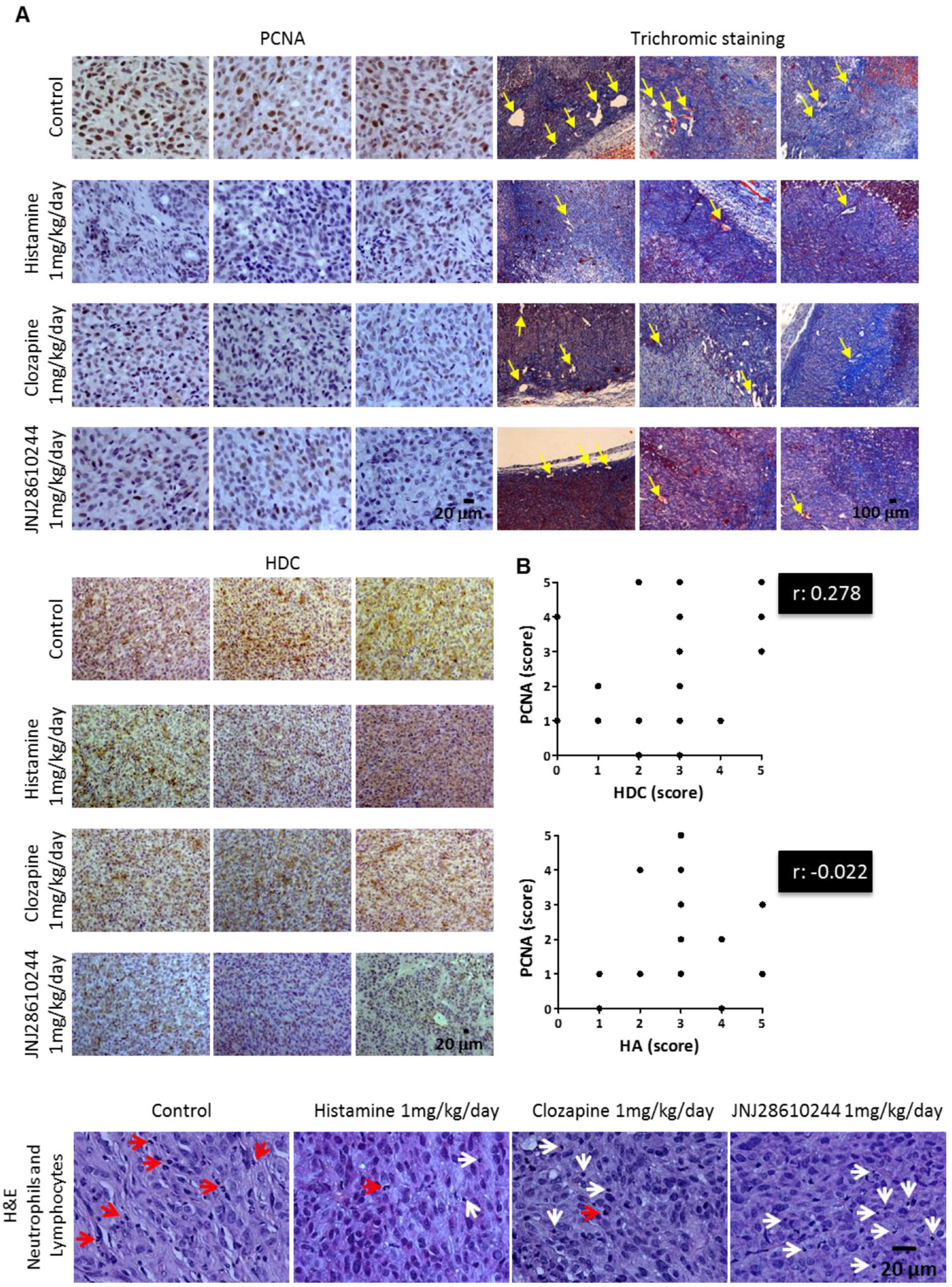

Supplementary Figure 2: (**A**) Complementary microphotography of Figure 3. Antitumoral effect of H_4R agonists in 1205Lu xenografted tumor induced in nude mice. Formalin-fixed paraffin embedded tissue sections of control, histamine, clozapine, and JNJ28610244 mice were stained to evaluate intracellular levels of histamine, histidine decarboxylase (HDC), proliferation (PCNA and MI) and vascular morphology. Yellow arrows indicate tumoral vessels. Red arrows indicate neutrophils. White arrows indicate lymphocytes. Pictures were taken at a 400X-fold magnification for PCNA immunostaining (Scale bar = 20 µm), 200X-fold magnification for HDC immunostaining and 50X-fold magnification for trichromic stain (Scale bar =100 µm). (**B**) Correlation analysis in melanoma biopsies. Spearman's correlation between HDC vs. PCNA (correlation coefficient rho, *r*: 0.278, *P* = 0.2492) and histamine vs. PCNA (correlation coefficient rho, *r*: −0.022, *P* = 0.9272). The analysis in malignant tissue between HDC or intracellular histamine and proliferation and prognostic markers, or against H_4R expression levels showed no significant correlations.

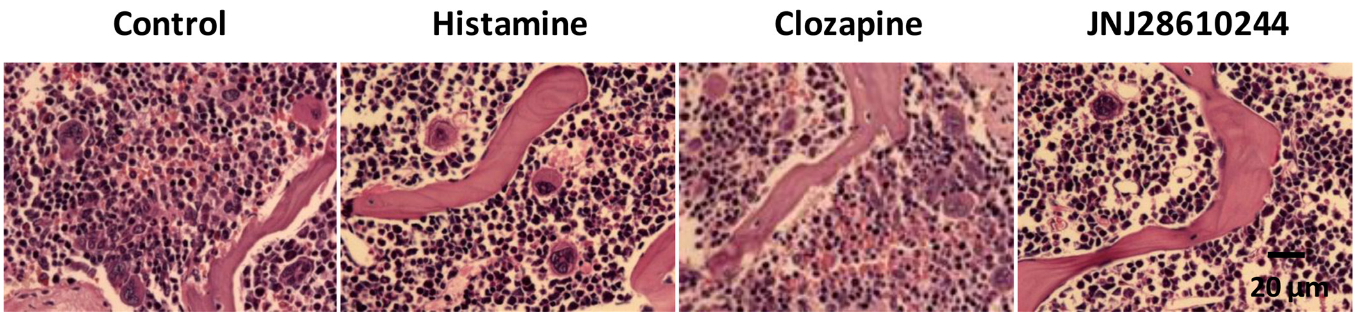

Supplementary Figure 3: Histological analysis of bone marrow. Regular trophism, normocellular and presence of three hematopoietic series (lymphoid, erythroid and myeloid) are observed. Stroma was unaltered in all cases. Pictures were taken at 400X-fold magnification. Scale bar = 20 µm.

A

Group	2 Gy SF
Control	0.39±0.02
RαMeH	0.21±0.03*
JNJ28	0.40±0.03
VUF	0.45±0.02

B

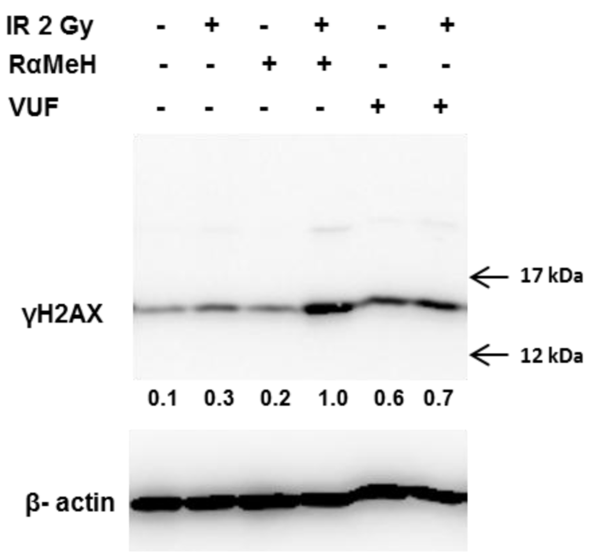

Supplementary Figure 4: Radiosensivity of 1205Lu melanoma cells. (**A**) 1205Lu cells were cultured in presence or absence of R-α-Methylhistamine (10 µM, RαMeH), JNJ28610244 (10 µM, JNJ28) or VUF8430 (10 µM, VUF) and radiobiological response was determined. 2Gy SF: fraction of surviving cells after exposure to 2 Gy dose. Survival curves were adjusted to the linear quadratic model [SF= e-(αD+βD2)]. Data represent the means ± SEM (*$P < 0.05$ vs. Control. *T*-test). (**B**) γH2AX (15 kDa) was assayed by Western blot. β-actin (42 kDa) was used as loading control. Semi-quantitative analyses of band intensities are shown ($n = 2$).

Supplementary Table 1: Primers and conditions used in RT-PCR

Gen	Primer F′<br>Primer R	Cycle N°	T (°C)	fragment (pb)	NCBI Reference Sequence
H_4R	GGG GTC TTG AAG ATT GTT AC<br>GCA GTT CAA CAT GTT CCC	35	57	512	NM_021624
β-actin	ACC TCA TGA AGA TCC TGA C<br>ACT CCT GCT TGC TGA TCC	25	58	521	NM_001017992
TYR	TTG GCA GAT TGT CTG TAG CC AGG<br>CAT TGT GCA TGC TGC TT	35	55	284	NM_000372

The PCR conditions were: H_4R 35 cycles of 45 s at 94°C; 45 s at 58°C; 50 s at 72°C [33]; TYR: 35 cycles of 1 min at 95°C; 1 min at 55°C; 1 min at 72°C [75]. β-actin: 25 cycles of 30 s at 95°C, 30 s at 58°C, 60 s at 72°C [71].

Supplementary Table 2: Main characteristics of human biopsies diagnosed with malignant melanoma (MM)

Clinical classification	Sex[a]	Clark[b]	Breslow[c]	Melanotic[d]	Amelanotic[d]	Stage[e]
Superficial spreading MM	F	I	ND		x	0
Superficial spreading MM	F	III	0.9 mm	x		II
Superficial spreading MM	F	I	ND	x		I
Superficial spreading MM	F	I	ND	x		I
Acral lentiginous MM	F	IV	2.8 mm	hipo		II
Acral lentiginous MM	F	II	6 mm		x	II
Nodular MM	M	IV	3 mm	hipo		II
Nodular MM	M	IV-V	15 mm	x		II
Nodular MM	F	V	10 mm	x		II
Nodular MM	F	IV	17 mm	hipo		II
Nodular MM	M	III-IV	3.5 mm		x	II
Nodular MM	ND	III	ND	x		II
Nodular spitzoid MM	M	III	1.1 mm	x		II
Nodular genital MM	M	III-IV	5.5 mm	x		II
Cerebral metastasis	M					IV
Parcial metastasis in centinel node	F					III
Subcutaneus metastasis	M					III
Subcutaneus metastasis	F					III
Masive metastasis in lymph node	M					III

[a] Patient sex: Female (F), Male (M); [b] Clark level (I–V); [c] Breslow thickness (mm); [d] Melanin content (Melanotic, Amelanotic); [e] Stage Disease (0–IV). The patients' age cannot be reported because it was not registered on the medical records. ND: Not determined.
